# NRF2 activation is required for chemotherapy resistance acquisition in medulloblastoma via metabolic and redox adaptation

**DOI:** 10.1016/j.redox.2026.104380

**Published:** 2026-09-03

**Authors:** Lorenzo Manfreda, Fatlum Rruga, Arianna Zanolli, Bryan P. Marzullo, Lisa Vettore, Martina Mozzo, Martina Canton, Chiara Marchioro, Elena Rampazzo, Elena Mariotto, Serena Filiberti, Marta Turati, Roberto Ronca, Daniel A. Tennant, Luca Persano, Giampietro Viola, Roberta Bortolozzi

**Affiliations:** a Department of Women's and Children's Health, University of Padova, Via Giustiniani 3, Padua, 35128, Italy; b Pediatric Research Institute, Corso Stati Uniti 4, Padua, 35127, Italy; c Department of Pharmaceutical and Pharmacological Sciences, University of Padova, Via Marzolo 5, Padua, 35131, Italy; d School of Medical Science, College of Medicine and Health, University of Birmingham, Birmingham, B15 2TT, United Kingdom; e Department of Molecular and Translational Medicine, University of Brescia, via Branze 39, Brescia, 25123, Italy

**Keywords:** NRF2 signaling, Redox homeostasis, Medulloblastoma, Chemotherapy resistance, Ferroptosis resistance

## Abstract

This study explores the pivotal role of NRF2 signaling in conferring resistance to chemotherapy and ferroptosis in medulloblastoma (MB), a highly malignant pediatric brain tumor. Using newly developed *in vitro* models of MB cells, resistant to standard chemotherapeutics (vincistine, etoposide, cisplatin, and cyclophosphamide), we observed that chemotolerant cells exhibit an enhanced antioxidant response. Specifically, we found higher levels of glutathione, compared to sensitive cells, and increased thioredoxin reductase activity, both key components in maintaining redox homeostasis. Furthermore, we identified a metabolic shift in resistant cells, marked by increased flux through the pentose phosphate pathway (PPP), which boosts NADPH production and supports the antioxidant defense mechanisms. This adaptive antioxidant response is largely mediated by hyperactivation of the NRF2 transcription factor and the consequent upregulation of a set of antioxidant genes, thus effectively reducing intracellular reactive oxygen species (ROS) levels and enhancing cells' ability to tolerate oxidative stress. Intriguingly, our results suggest that NRF2 activation not only supports the acquisition of chemotherapy resistance but also confers protection against ferroptosis induction. Indeed, resistant cells upregulate iron sequestration proteins and ferroptosis-suppressing genes, directly controlled by NRF2, thereby reducing vulnerability to lipid peroxidation-induced cell death. Inhibition of NRF2 in resistant cells increased their sensitivity to both chemotherapy and ferroptosis inducers and, more interestingly, its knockdown prevented sensitive cells from acquiring resistance. Overall, our study underscores the crucial role of NRF2-controlled redox homeostasis in mediating resistance mechanisms in MB, highlighting the therapeutic potential of targeting this pathway. Targeting NRF2 signaling may provide a novel approach to overcome therapy resistance and improve treatment outcomes for patients with this challenging cancer.

## 1 Introduction

Medulloblastoma (MB) is one of the deadliest brain tumors of childhood, which occurs in the posterior fossa and likely arises from embryonic neural cells harboring gene and pathway abnormalities involved in the maintenance of cell pluripotency [1]. MB is intrinsically characterized by rapid growth, high invasiveness, and resistance to treatments. Indeed, MB patients frequently experience tumor relapse and, despite an intensive therapeutic regimen including a combined approach of surgery, radio, and chemotherapy, tumor recurrence is estimated to occur in 30-40% of cases. Further treatments are generally ineffective, with poor outcomes in terms of overall survival [2]. One of the major causes of treatment failure depends on the development of chemotherapy resistance, which arises in MB irrespective of genotype, possibly as an adaptive response to the treatment pressure [3]. Changes in cell phenotype strongly support the hypothesis that MB tumors are non-static entities but rather evolve under the pressure of microenvironmental cues and drug treatments [4]. In this context, understanding the molecular mechanisms underlying therapy resistance is crucial for advancing treatment strategies, particularly in the MB field, which is further complicated by the significant heterogeneity in the genetic/genomic and transcriptomic landscape of the disease [[5], [6], [7]].

Considering that the study of chemotherapy resistance and tumor recurrence in MB is limited by the poor availability of matched diagnosis-relapse samples, our research group recently developed and characterized new *in vitro* models of MB cells resistant to the key chemotherapeutics clinically used for MB management: vincristine, etoposide, cisplatin, and cyclophosphamide (VECC) [8,9]. The molecular profiling of these resistant cells revealed significant metabolic remodeling, characterized by enhanced antioxidant response and improved detoxification activity within the cells [8].

In recent years, the control of redox homeostasis has emerged as a critical hallmark in cancer biology since the tight regulation of reactive oxygen species (ROS) levels in cancer cells contributes to their survival [10]. Cancer cells typically exhibit elevated ROS levels compared to normal cells as a consequence of altered metabolism, genomic instability, mitochondrial dysfunction, and tumor microenvironment [11]. In addition, an appropriate oxidative balance is essential for cancer progression, since it stimulates tumorigenesis and can control different cancer cell behaviors, including migration and cell proliferation. Conversely, excessive ROS accumulation could be detrimental to cancer cells. Hence, they abnormally upregulate antioxidant systems to keep ROS levels within a favorable range, in order to prevent oxidative damage and thus escape from cell death [12]. Most chemotherapeutics, including vincristine, cisplatin, etoposide, and cyclophosphamide, have been demonstrated to have potent anticancer activity by inducing ROS accumulation and oxidative stress [[13], [14], [15]]. Moreover, emerging studies highlight the relevance of metabolic plasticity and the upregulation of antioxidant systems in promoting chemotherapy resistance and adaptation to both intrinsic and drug-induced oxidative stress [16,17]. In this context, the NRF2 signaling pathway is one of the most important systems developed against oxidative stress and toxic xenobiotic insults in mammalian cells. NRF2 controls the expression of approximately 200 genes, mostly involved in drug detoxification (phase I/II/III drug metabolism), redox homeostasis (glutathione and thioredoxin-based antioxidant systems), and heme and iron metabolism. In addition, NRF2 enhances NADP^+^-reducing enzymes such as glucose-6-phosphate dehydrogenase (G6PD), 6-phosphogluconate dehydrogenase (PGD), isocitrate dehydrogenase 1 (IDH1), and malic enzyme 1 (ME1), supplying cells with the necessary reducing power to counteract the excess of oxidative stress [18].

Despite many findings demonstrating the paramount role of NRF2 in protecting cells from cancer initiation, cardiovascular diseases, inflammation, and neurodegenerative diseases [[19], [20], [21]], increasing evidence has identified the NRF2 pathway as a driver of cancer progression [[21], [22], [23]]. Its increased activation has been observed in several human cancers and correlates with poor clinical outcomes, as it sustains cancer cell proliferation and promotes chemo- and radio-resistance [21,22,24]. Notably, it was recently demonstrated that NRF2 expression could be induced by certain anticancer agents, including vincristine, cisplatin, and etoposide as a pro-survival response against drug treatments [15,[24], [25], [26], [27]]. Furthermore, recent studies highlight a relevant role of NRF2 in regulating ferroptotic cell death through the control of the intracellular labile iron metabolism and the protection against lipid peroxidation [28,29].

In this study, we demonstrate that redox homeostasis is tightly regulated in MB chemotherapy-resistant cells through the increased activation of the NRF2 pathway, which is required for the acquisition of chemotherapy resistance. Our findings indicate that the activation of the NRF2 transcriptional activity, induced by chemotherapy itself, stimulates the cellular defense mechanism devoted to redox balance maintenance, enabling MB-cells to manage induced oxidative stress. In addition, contributing to their survival within the chemotoxic environment, NRF2 activation confers chemotherapy-resistant MB-cells protection against ferroptosis induction.

## 2 Materials and methods

### 2.1 Medulloblastoma cell cultures

Medulloblastoma DAOY and HD-MB03 cells were purchased from ATCC (Manassas, VA, USA) and grown in RPMI, supplemented with 10% fetal bovine serum (FBS, Thermo Fisher Scientific, Waltham, MA) and 1% penicillin/streptomycin (stock solutions: Penicillin 10000units/mL and Streptomycin 10 mg/mL; Thermo Fisher Scientific, Waltham, MA). Cells were maintained in a standard incubator (37°C in a humidified atmosphere with 5% CO2). HuTuP33 primary cultures were previously derived from a pediatric MB tumor taken at surgery. The initial pathological review was followed by a secondary neuropathological review to reconfirm the diagnosis. Written informed consent for the donation of tumor brain tissues was obtained from parents, before tissue acquisition, under the auspices of the protocol for the acquisition of human brain tissues obtained from the Ethical Committee board of the University of Padova and Padova Academic Hospital. The tissue was acquired following the tenets of the Declaration of Helsinki. Tumor specimens were enzymatically and mechanically dissociated into single-cell suspensions, properly expanded, and grown as monolayers, as previously described [30]. HuTuP33 were cultured on fibronectin-coated dishes in DMEM/F12 medium (Biowest, Nuaillé, France) supplemented with B-27 (Thermo Fisher Scientific, Waltham, MA) and 40 ng/ml basic Fibroblast Growth Factor (bFGF), 40 ng/ml Epidermal Growth Factor (EGF; both from Cell Guidance Systems Ltd, Cambridge, UK) and 0.5% penicillin/streptomycin (Gibco, Waltham, MA USA). Primary cells were maintained in a H35 hypoxic chamber (Don Whitley Scientific Ltd, Shipley, UK) with 1.5% oxygen, 5% carbon dioxide, and balance nitrogen.

Chemotherapy-resistant cells (DAOY-R, HD-MB03-R, and HuTuP33-R cells) were generated and characterized in our previous paper [8].

### 2.2 Reagents and drug treatments

RSL3, sulforaphane, and N-Acetyl-Cysteine (NAC) were purchased from Sigma-Aldrich (Burlington, MA, USA), whereas G6PDi, erastin, ferrostatin-1, enzastaurin, and ML385 were obtained from MedChemExpress (Monmouth Junction, NJ, USA). Cells were seeded in appropriate multiwell plates and allowed to attach for 24 h before treatment. Concentrations and exposure times are reported in the corresponding figure legends.

### 2.3 Proliferation inhibition assay

For antiproliferative assays, we employed resazurin, a fluorogenic cell-permeable dye that is used as a cell viability indicator. Briefly, cells were seeded in 96-well plates and after 24 h were treated with test compounds for 72 h. To quantify cell viability, resazurin (7-Hydroxy-3H-phenoxazin-3-one-10-oxide, Sigma Aldrich, Burlington, Massachusetts, USA) was added to each well (final concentration in cell media 10 μg/mL) and the plate was incubated for 2h in a standard incubator (5% CO2, 37°C, humidity 95%). Fluorescence was acquired using the Spark multimode plate reader (Tecan, Männedorf, Switzerland). In all graphs, data have been expressed as % of cell viability relative to control cells.

### 2.4 TxR activity

TxR activity was measured with Thioredoxin Reductase Colorimetric Assay Kit (Cayman Chemical, USA). In particular, this enzymatic assay is based on the reduction of DTNB (5,5′-dithio-bis(2-dinitrobenzoic acid, Ellman's Reagent) with NADPH to 5- thio-2-nitrobenzoic acid (TNB).

According to manufacturer protocol, we collected total proteins from DAOY-S/-R and HD-MB03-S/-R under basal conditions by homogenizing each cell pellet in lysis buffer containing 50 mM potassium phosphate and EDTA 1 mM (pH 7.4). After centrifuging samples at 10000 x g for 15 min at 4°C, we collected supernatants. To measure TxR activity in each sample, 20 μL of each lysate was mixed with NADPH and DTNB. Absorbance at 405 nm was detected once every minute for at least 5 time points. Data were normalized on total protein quantification, measured in each lysate using the Pierce BCA Protein Assay Kit (Thermo Fisher Scientific, Waltham, MA, USA).

### 2.5 G6PD activity

Glucose-6-phosphate dehydrogenase (G6PD) activity was evaluated using the Glucose-6-Phosphate Dehydrogenase Activity Assay Kit (Cayman Chemical, Michigan, USA). In this assay, the ability of G6PD to catalyze the oxidation of glucose-6-phosphate to 6-phospho-d-gluconate with a concomitant reduction of NADP ^+^ to NADPH is measured through the reaction of NADPH with a fluorimetric detector. 10^6^ DAOY-S/-R and HD-MB03-S/-R were collected under basal conditions. After sonication (1 ml of cold PBS) and centrifugation (10000 g, 10 min at 4°C), supernatants were collected and used for the assay, following the manufacturer's instructions. The assay was performed at 37°C, and fluorescence was monitored at λex 535 and λex 585. Results were normalized based on the total protein concentration of each sample.

### 2.6 Western blot

Total cell lysates were prepared in MPER (Pierce, Waltham, MA, USA) lysis buffer containing Halt™ Protease and Phosphatase Inhibitor Cocktail (Pierce, Waltham, MA, USA), while nuclear and cytoplasmic protein fractions were isolated using Nuclear/Cytosol Fractionation Kit (Abcam, Waltham, MA, USA). Equal amounts of total protein lysates (10-20 μg) were resolved using SDS-PAGE gels (NuPage; Thermo Fisher Scientific, Waltham, MA). Proteins were transferred to PVDF Immobilon-P Membrane (Merck Millipore, Burlington, Massachusetts, USA) and incubated for 2 h in 3% bovine serum albumin (BSA, Applichem GmbH, Darmstadt, Germany). Membranes were incubated with primary antibodies (Table 1) under constant shaking overnight at 4°C. Membranes were next incubated for 1 h with HRP-conjugated secondary antibodies (Revvity, Waltham, Massachusetts, USA), developed using ECL Select reagent (Cytiva, Marlborough, MA), and visualized using the iBright FL1500 Imager (ThermoFisher, Waltham, MA, USA). To ensure equal protein loading, each membrane was stripped and reprobed with an anti-vinculin or anti-β-actin antibody. Image analysis and band quantification were performed using ImageJ software. Band intensities were quantified and normalized to the corresponding loading control for each sample.

**Table 1:** Antibodies used in immunoblot analysis.

NRF2 s40	Novus Biologicals	NBP2-67465
NRF2	Novus Biologicals	NBP1-32822
NRF2	Abcam	ab62352
Tkt	Santa Cruz Biotecnology	sc-390179
Nqo1	Santa Cruz Biotecnology	sc-393736
Vinculin	Santa Cruz Biotecnology	sc-73264
G6PD	Santa Cruz Biotecnology	sc-373886
PRDXIII	Genetex	GTX112004
SOD2	Santa Cruz Biotecnology	sc-133134
PKCα (s657)	Millipore	06 822
PKCα	Upstate	#05-154
GAPDH	Genetex	GTX627308
Histone H3	Cell Signaling	#9715
FSP1	Proteintech	68049-1-Ig
SLC7A11	Proteintech	26864-1-AP
β-Actin	Sigma Aldrich	A5316

### 2.7 Luciferase reporter assay

Cells were seeded in a 6-well plate (70 000 cells/well for DAOY cells, 200 000 cell/well for HD-MB03 and HuTuP33 cells) and after 24 h were transiently transfected using TransIT® -LT1 Transfection Reagent (Mirus Bio LLC, Madison, WI, USA) with 2.5 μg of ARE-luciferase reporter construct PGL-8xARE plasmid (ARE-luc) [15] together with the 1 μg of pMAX-GFP plasmid for normalization purposes. The ARE-luc plasmid, kindly provided by Prof. C. Roland Wolf, was obtained by the insertion of 8 copies of ARE sequences upstream the firefly luciferase gene [15]. After 48h, firefly luciferase activity was measured using Luciferase Assay Substrate and Buffer from Promega (Madison, WI, USA), while GFP-fluorescence was detected as transfection control (λex 485 nm/λem 535 nm). Data were acquired using the Spark multimode plate reader (Tecan, Männedorf, Switzerland). Values, expressed as relative light units (RLUs), were normalized to GFP fluorescence values in order to correct for possible heterogeneity in transfection efficiency.

### 2.8 NRF2 gene silencing by short hairpin RNAs and small interfering RNA

For the inducible knockdown of *NFE2L2*, HD-MB03-R cells were transduced with lentiviral particles allowing for the doxycycline-dependent expression of a shRNA targeting *NFE2L2* (tet-pLKO.1-puro-shNRF2) (Addgene #136584) or the firefly luciferase sequence (tet-pLKO.1-puro-shLUC) (Addgene #136587), as control. HD-MB03 cells were infected with MOI 10 and selected in puromycin (10 μg/ml). In the experiments, NRF2 silencing was induced by treating cells with doxycycline (1 μg/ml) for 48 h before use. In some experiments, DAOY-S/R and HD-MB03-S/R cells were transduced (MOI 10) with a lentiviral vector stably expressing a short hairpin against NRF2 (shNRF2) or a non-targeting sequence as control (shNT) (VectorBuilder GmbH, Germany).

Transient knockdown of NFE2L2 was performed with an NFE2L2-specific siRNA (Santa Cruz Biotechnology, Dallas, Texas, USA) using the siRNA Transfection Reagent (Santa Cruz, sc-29528) for DAOY cells or using the Transit-X2 Transfection Reagent (Mirus Bio LLC, Madison, WI) for HuTuP33 cells following the manufacturer's instructions. A non-targeting siRNA (siNEG) (Santa Cruz Biotechnology, Dallas, Texas, USA) was used as a proper control.

### 2.9 Cytofluorimetric analysis

Intracellular ROS levels were detected by the 2,7-dichlorodihydrofluorescein diacetate (H2-DCFDA; Molecular Probes, Eugene, Oregon, USA) dye. Cells were stained for 30 min in 20 μM 2,7-Dichlorodihydrofluorescein diacetate (H2-DCFDA) at 37°C, in the dark.

Reduced glutathione levels were detected by the monobromobimane probe (MBB; Molecular Probes, Eugene, Oregon, USA) by incubating cells with 50 μM MBB for 30 min at 37°C in the dark.

Apoptosis was evaluated by using Annexin V staining (Molecular Probes, Eugene, Oregon, USA). Briefly, VECC-treated cells were incubated with Annexin V-FITC and propidium iodide at room temperature for 15 min, then fluorescence was acquired at 530 nm and 575 nm using 488-nm excitation.

Data were acquired using the CytoFLEX flow cytometer (Beckman Coulter, Brea, CA, USA) and were analyzed using FlowJo software v.10.7.1 (BD Biosciences, Franklin Lakes, NJ, USA).

### 2.10 Detection of intracellular Fe^2+^

HD-MB03 cells were plated in 96-well cell culture/imaging plates (Agilent Technologies, Santa Clara, CA, USA) at a density of 2 × 10^4^ cells per well. 24 h after seeding, cells were incubated with 1 μM BioTrackerTM FerroOrange Live Cell Dye (Merck Millipore, Burlington, Massachusetts, USA) in HBSS at 37°C for 30 min. After the staining, cells were washed twice in HBSS and analyzed with a Zeiss LSM 800 Confocal Spectral microscope (Zeiss, Oberkochen, Germany). Mean Fluorescence Intensity (MFI) was measured using Fiji ImageJ.

### 2.11 ROS generation kinetics

An equal number of cells were seeded in triplicate for each condition in a white 96-well plate. The day after, cells were stained with 10 μM H2-DCFDA for 30 min at 37°C. Excess H2-DCFDA solution was removed, and cells were washed 2 times with Hank's balanced salt solution. Control or hydrogen peroxide-containing media (250 μM) were added to the cells, and then H2-DCFDA-associated fluorescence intensity was monitored over time in the Spark multimode plate reader (Tecan, Männedorf, Switzerland) with an excitation wavelength of 485 nm and emission of 535 nm.

### 2.12 Immunofluorescence

Cells were seeded in a cell culture/imaging microplate (Agilent Technologies, Santa Clara, CA, USA) After 24 h, cells were fixed with 4% formaldehyde (Carlo Erba), permeabilized, and blocked with a solution containing 3% Bovine Serum Albumin (BSA) and 0.1% Triton X100 (both from Applichem GmbH, Darmstadt, Germany) at room temperature. Next, cells were incubated overnight at 4°C with an anti-NRF2 antibody (Novus Biologicals, Colorado, USA). Cells were then stained with AlexaFluor488-conjugated secondary antibody for 1 h at room temperature in the dark. Nuclei were briefly counterstained with Hoechst33342 (ThermoFisher, Waltham, MA, USA). Images were acquired through the Zeiss LSM 800 Confocal Spectral microscope (Zeiss, Oberkochen, Germany). Mean Fluorescence Intensity (MFI) was measured by using Fiji ImageJ.

### 2.13 Gene expression data analysis

Transcriptional data of MB-S and MB-R were obtained from previously published gene expression data from our group (Series Accession Number GSE220543) [8] and analyzed for the enrichment of REACTOME_KEAP1_NFE2L2_PATHWAY using GSEAv2.0.

**Fig. 1:**
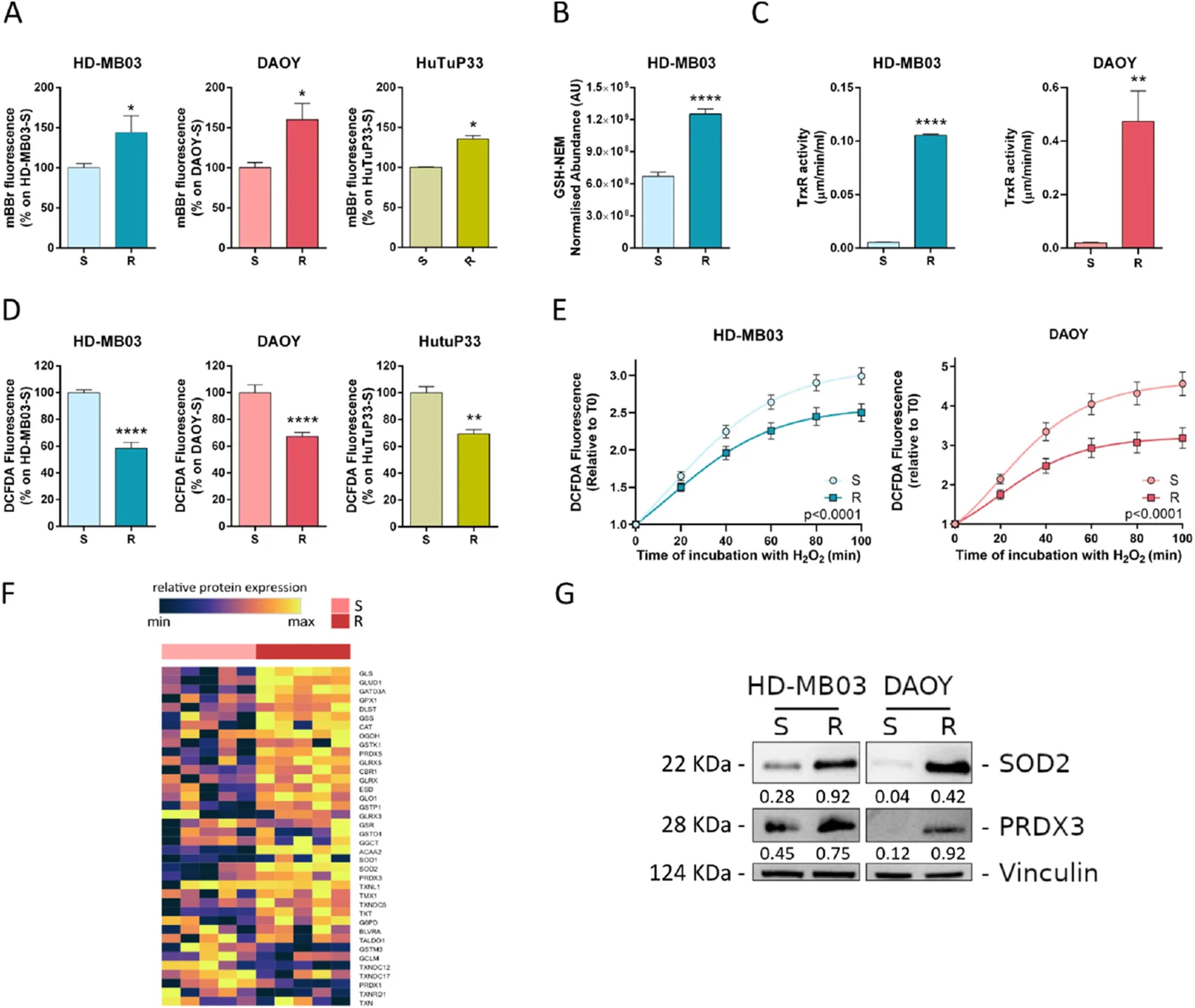
A Flow-cytometry-based monobromobimane (mBBr) quantitation of GSH in MB-R and MB-S cells as indicated. Data are represented as the percentage of mean fluorescence intensity (MFI) of the dye over MB-S cells. **B** Normalized abundance of GSH (as its NEM-derivative) in HD-MB03-S and HD-MB03-R cells measured by LC/MS. **C** TxR activity was measured as the NADPH-dependent production of 5-thio-2-nitrobenzoic acid (TNB) per minute. **D** The amount of cellular ROS was measured by flow cytometry using DCFDA and represented as the percentage of the MFI of oxidized dye relative to MB-S cells**. E** ROS levels during oxidative stress by incubation with hydrogen peroxide (500 μM) were measured over time (100 min) in HD-MB03-S/R and DAOY-S/R cells. Following the staining with H2-DCFDA probe, hydrogen peroxide was added, and the fluorescence of the oxidized dye was recorded from time 0 every 20 min. **F** Heatmap reporting normalized protein abundance of differentially expressed proteins (*p* < 0.05, n = 38 by Holm–Šídák multiple *t*-test) related to antioxidant defense systems between DAOY-S (pink) and DAOY-R (red) samples, 5 biological replicates were evaluated for each condition. For visualization, expression values were normalized using min–max scaling for each gene, setting the minimum and maximum expression values to 0 and 1. Colors indicate relative expression levels. **G** Western Blot analysis of SOD2, PRDX3, and vinculin as a loading control, in HD-MB03 and DAOY sensitive and resistant cells.

The heatmap in Fig. 1 was generated by the Morpheus bioinformatic tool (https://software.broadinstitute.org/morpheus/). The *NRF2*^high^ signature was generated by using the core enrichment genes, which contribute to the REACTOME_KEAP1_NFE2L2_PATHWAYenrichment by comparing MB-R vs MB-S cells (Supp Table S1). We then evaluated the prognostic potential of this signature in a publicly available MB patient transcriptional dataset (GSE85217) [7]. The log2 expression values for each patient sample in the dataset were centered at zero mean. The sum of the mean-centered log2 expression values of the NRF2^high^ probe sets was then used as the Risk Score for each subject and the 763 subjects from GSE85217 were split into high- and low-risk groups greater and less than the median risk score, respectively [31]. These risk groups were assessed for prognostication of OS in univariate Cox analysis (log-rank test).

### 2.14 Reverse transcription and quantitative real-time (RT) PCR

Total cellular RNA from cell lines and patient bone marrow was extracted using the miRNeasy Micro Kit (Qiagen, Hilden, Germany) according to the manufacturer's protocol. For cDNA synthesis, 1 μg of RNA was reverse-transcribed using SensiFAST™ cDNA Synthesis Kit (Meridian Biosciences, USA). Quantitative real-time PCR was performed using SensiFAST™ SYBR® Lo-ROX Kit (Meridian Biosciences, USA) and analyzed on QuantStudio 5 Real-Time PCR Systems (Thermo Fisher Scientific, Waltham, MA, USA). The oligonucleotides used for PCR amplification are listed in Table 2. Expression values were normalized to GUSB according to the ΔΔCt method. Data are reported as log2 fold change expression of genes of interest, as indicated in the histograms.

**Table 2:** Primer sequences used for real-time PCR amplification.

Gene	Forward sequence (5′-3′)	Reverse sequence (5′-3′)
NFE2L2	GAGAGCCCAGTCTTCATTGC	TGCTCAATGTCCTGTTGCAT
FTH1	GCCAGAACTACCACCAGGACT	CAGCATGTTCCCTCTCCTCAT
NQO1	GGGCAAGTCCATCCCAACTG	GCAAGTCAGGGAAGCCTGGA
GUSB	GAAAATACGTGGTTGGAGAGCTCATT	CCGAGTGAAGATCCCCTTTTTA

### 2.15 Metabolomics and metabolic tracing using LC/MS

For LC/MS steady-state metabolomics and tracing experiments, cells were seeded at a concentration of 250.000 cells/60 mm petri dishes (for DAOY) or 250.000/well of 6-well plates (for HD-MB03) in complete RPMI with dialyzed FBS or, for isotope tracing, RPMIØ+Glucose where glucose was replaced by its isotopically labeled analogue ^13^C-[U]-Glucose. After 24 h, cells were harvested using a cell scraper, washed with 200 μl of ice-cold PBS, and metabolites extracted using 100 μl of ice-cold precipitation mix (acetonitrile, methanol, water (40:40:20) with 0.5% formic acid) containing 0.25 μM mercaptopurine and 1 μg/ml D6-glutaric acid as internal standards. Samples were vortexed and incubated on ice for 5 min. The extraction mixture was then neutralised using 15% ammonium bicarbonate solution (8.8 μL), vortexed, and ice-chilled for a further 15 min. Following centrifugation (20 000 g, 4°C for 10 min), the supernatant was transferred to a new tube and stored at −80°C before analysis.

For the analysis of GSH, cells were seeded as described above in RPMI (dialyzed FBS). After 24h, cells were collected and washed with 200 μl of ice-cold PBS/NEM solution (6.67 mM NEM in PBS). Metabolites were extracted using 100 μl of ice-cold precipitation mix (6.67 mM NEM in 50:50 acetonitrile/methanol with 1 μg/ml D6-glutaric acid as internal standard). Before analysis, samples were thawed in ice and transferred into LC-MS vials. Samples were loaded into a temperature-controlled sample manager set at 4°C. For the steady-state metabolomics and tracing experiments, 4-6 μl were injected into an Agilent 1290 Infinity II Liquid Chromatography system equipped with an Atlantis Premier BEH Z-HILIC 1.7 μm, 2.1 × 150 mm column (Waters) and a Vanguard precolumn. Solvent A consisted of 20 mM Ammonium Bicarbonate (LiChropur, Sigma) with 0.1% v/v Ammonium Hydroxide (Alfa Aesar) in 100% water (Honeywell), containing 5 μM InfinityLab Deactivator Additive Solution (Agilent). Solvent B was a 90:10 mixture of acetonitrile and water, also containing 5 μM InfinityLab Deactivator Additive Solution. A non-linear binary gradient was employed as follows: 0 min 85% B, 2 min 85% B, 18 min 60% B, 22 min 30% B, 22.1 min 10% B, 25 min 10% B, 25.1 min 85% B, and 30 min 85% B. The column compartment was maintained at 30°C. Blank runs were included every 6–9 samples to monitor metabolite carryover. GSH-NEM was analyzed on a Waters Xevo TQ-XS using a Acquity Premier BEH Amide 2.1 × 100mm column held at 30°C. The mobile phase A consisted of 20 mM Ammonium Bicarbonate with 0.1% v/v Ammonium Hydroxide in 100% water. Mobile phase B was 90:10 acetonitrile and water. The gradient used was as follows: 0 min 95% B, 0.5 min 95% B, 1 min, 94.5% B, 1.5 min 94% B, 2 min 87% B, 2.5 min 85% B, 3 min 82% B, 4 min 80% B, 6.5 min 65% B, 7 min 90% B, 7.5 min 95% B, and 10 min 95% B. The resulting data were analyzed using Skyline (MacCoss Lab Software) and normalized using in-house R scripts.

An Agilent 6546 Q-TOF mass spectrometer was used for metabolite and isotopologue measurements, operating in high-resolution mode with single-polarity negative electrospray ionization. Mass calibration was performed before each sample batch. The drying gas flow rate was 8 l/min at 225°C, the nebulizer pressure was 30 psi, and the sheath gas temperature was 300°C with a flow rate of 12 l/min. The VCap was set at 2000V, with a 500V nozzle voltage, a fragmentor voltage of 125V, a skimmer voltage of 60V, and an octopole RF voltage of 750V. MS1 scans were performed at 1Hz across a range of 50–1050 m/z. Real-time mass correction was achieved using continuously infused reference masses of 112.985587 and 1033.988109. Raw data files (.d) were imported into Agilent MassHunter Profinder for batch isotopologue extraction using an in-house Personal Compound Database and Library (PCDL). This library included metabolite IDs validated by MS/MS and pure chemical standards for isotope tracing. A 10 ppm mass tolerance was applied, and all potential metabolite or isotopologue peaks were manually reviewed for accuracy. Data were normalized to internal standards and cell counts using custom scripts in Excel (Microsoft).

#### 2.15.1 Sulforhodamine B staining

HD-MB03-R shNT and shNRF2 cells were plated in 24-well plates at a density of 5 × 10^4^ cells/well. After 24 h, cells were exposed to VECC at the indicated concentrations. Following 72 h of treatment, cells were fixed with ice-cold 10% (w/v) trichloroacetic acid (Sigma-Aldrich, Burlington, Massachusetts, USA) and incubated overnight at 4°C. Cells were subsequently washed with distilled water, air-dried at room temperature, and stained with 0.4% (w/v) sulforhodamine B (SRB) (MedChemExpress, Monmouth Junction, NJ, USA) solution for 30 min. Excess dye was removed by repeated washes with 1% (v/v) acetic acid. Following solubilization of the protein-bound SRB dye in 10 mM Tris base solution (Carlo Erba, Milano, Italy), the absorbance was measured at 510 nm using a Spark multimode microplate reader (Tecan, Männedorf, Switzerland).

### 2.16 In vivo models

Animal Experiments were performed according to the Italian laws (D.L. 116/92 and following additions) that enforce the EU 86/109 Directive and were approved by the local animal ethics committee (OPBA, Organismo Preposto al Benessere degli Animali, Università degli Studi di Brescia, Italy). For the subcutaneous xenograft model, 7-week-old NOD/Scid mice were injected subcutaneously into the flank with 3 × 10^6^ human MB cells (HD-MB03-shNT and HD-MB03-shNRF2 cells. Tumors were measured with a calliper, and the volume was calculated according to formula V = (D × d^2^)/2, where D and d are the major and minor perpendicular tumor diameters, respectively. When tumors reached the dimension of ≈ 50 mm^3^ animals were randomized and the first treatment started injecting i.p. vehicle (control) or VCC (cisplatin 2.5 mg/kg + vincristine 0.1 mg/kg on the first day and cyclophosphamide 75 mg/kg on the second day). The second treatment (both control and VCC) was administered 12 days after. Tumor growth was followed and at the end of the experimental procedure, tumors were surgically removed, weighed and snap frozen.

### 2.17 Statistical analyses

Graphs and associated statistical analyses were generated using GraphPad Prism 10.4.2 (GraphPad, La Jolla, CA). All data in bar graphs are presented as mean ± standard error of the mean (S.E.M.). Statistical significance between two groups was measured by paired *t*-test, and multiple-group comparisons were analyzed by one-way ANOVA. *p < 0.05, **p < 0.01, ***p < 0.001,****p < 0.0001. Heatmaps and level plots displayed in figures have been generated through the Morpheus open access platform (https://software.broadinstitute.org/morpheus).

## 3 Results

### 3.1 MB-resistant cells enhance oxidative stress detoxification systems

Multiomic analysis previously performed by our group on chemotherapy-resistant counterparts of HD-MB03 and DAOY cell lines and HuTuP33 patient-derived cancer cells suggests that chemoresistant MB cells (MB-R) enhance antioxidant response systems and improve their ability to maintain redox homeostasis [8].

To gain insight into the redox status of chemotherapy-resistant cells, we evaluated in matched sensitive (S) and resistant (R) HD-MB03, DAOY, and HuTuP33 MB cell models the activation status of the glutathione (GSH) and thioredoxin (Trx) scavenging systems, the two major thiol-dependent antioxidant systems activated by mammalian cells to counteract oxidative stress [32]. Cytofluorimetric analysis, using the thiol-reactive probe monobromobimane (mBBr), unveiled a significantly greater abundance of reduced thiols in MB-R cells, suggesting they possess an increased capacity of maintaining a reduced intracellular environment (Fig. 1A). Confirming these data, MS analysis showed that intracellular levels of GSH are significantly higher in HD-MB03 cells (Fig. 1B). In addition, we assessed the enzymatic activity of thioredoxin reductase (TxR), which catalyzes the NADPH-dependent reduction of the redox protein thioredoxin. Through a TxR enzymatic assay, we demonstrated that resistant cells are characterized by a significantly higher TxR activity in comparison to their sensitive counterparts, with 3- and 17-fold increases in the ability to reduce DTNB (5,5-dithio-bis-(2-nitrobenzoic acid)) in DAOY-R and HD-MB03-R cells, respectively (Fig. 1C).

To evaluate whether the enhanced antioxidant capacity displayed by chemoresistant MB cells correlates with an elevated ability to buffer reactive oxygen species (ROS), we stained cells with H2-DCFDA, demonstrating that, under basal conditions, all tested MB-R cells accumulate significantly lower intracellular ROS than their relative parental (S) cells (Fig. 1D).

To functionally validate this observed increase in the ROS scavenging capacity, ROS were also monitored after acute induction of oxidative stress through hydrogen peroxide. We observed a significantly lower accumulation of ROS in DAOY-R and HD-MB03-R cells over time compared to their sensitive counterparts, further indicating that resistant cells are endowed with enhanced ability to attenuate oxidative stress (Fig. 1E).

Altogether, these results demonstrate that MB-R cells exhibit a significantly enhanced antioxidant capacity, characterized by increased levels of reduced thiols, elevated glutathione (GSH) content, and greater thioredoxin reductase (TxR) activity.

To better account for the potential proteomic remodeling able to sustain redox homeostasis in chemoresistant MB cells, we re-analyzed our previously published proteomics data [8] by focusing on redox-related proteins, intriguingly finding a general overexpression of relevant proteins controlling ROS homeostasis in cells. (Fig. 1F–Table S2). Importantly, we validated our proteomic analysis by analyzing the expression levels of SOD2 and PRDX3, two mitochondrial proteins that regulate the conversion of superoxide (O2^•-^) into hydrogen peroxide (H_2_O_2_) or oxygen (O_2_) (depending on the oxidative state of manganese required for its function), and of hydrogen peroxide into water (H_2_O), respectively. Both enzymes showed higher expression levels in MB-R cells (Fig. 1G), confirming their potential contribution in supporting enhanced ROS detoxification in chemotolerant cells.

Indeed, these potentiated redox homeostasis systems enable resistant cells to maintain a reduced intracellular environment and more effectively buffer ROS. Of note, this happens both under basal conditions and during acute induction of oxidative stress, suggesting that improved oxidative stress detoxification plays a critical role in the survival and adaptation of MB-R cells during treatments.

### 3.2 Pentose phosphate pathway-dependent NADPH production fuels reducing capacity and sustains cell viability in MB-R cells

**Fig. 2:**
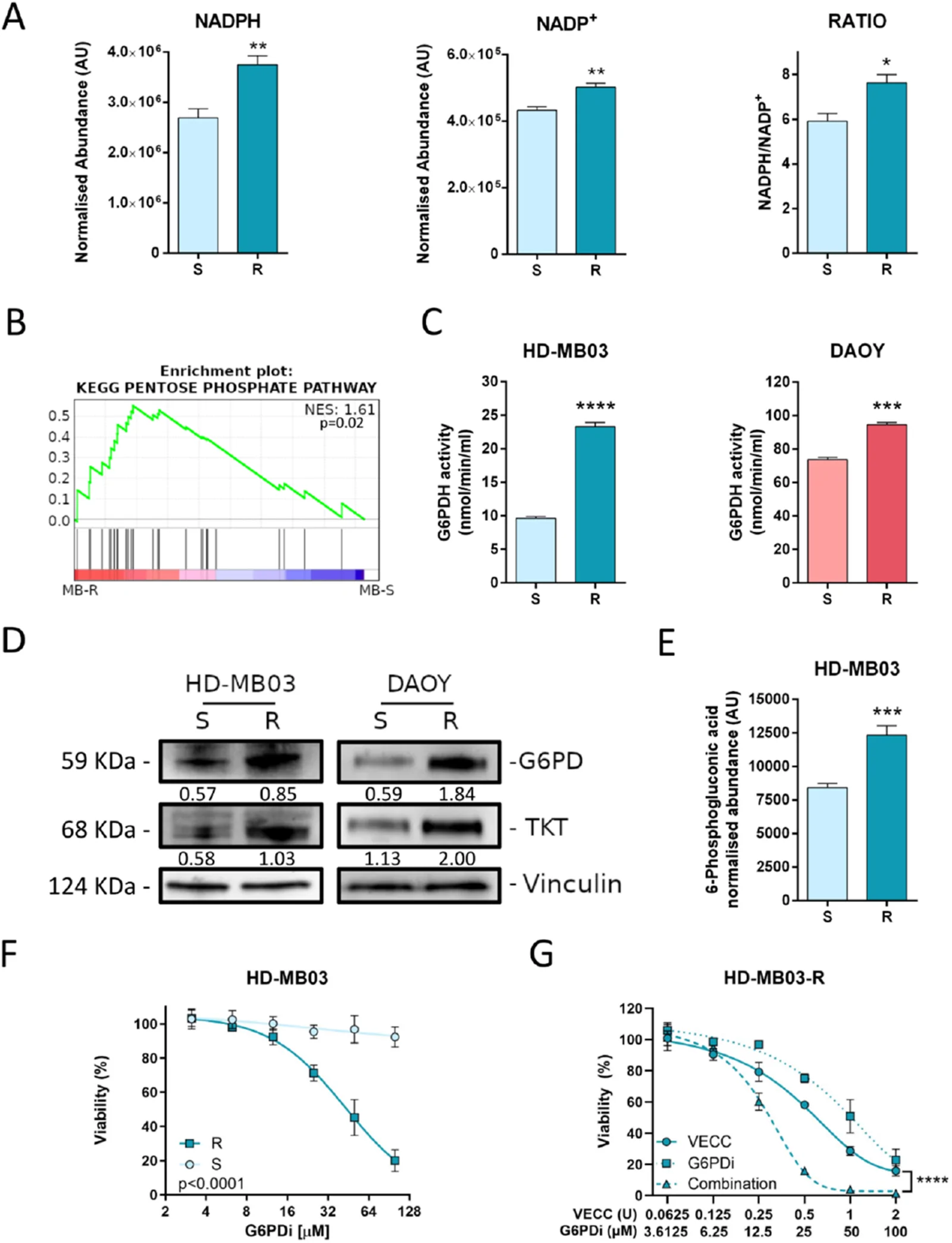
A Intracellular NADPH, NADP^+^ abundance, and their ratio in HD-MB03 cells. *p < 0.05, **p < 0.01 by multiple *t*-test comparison. **B** GSEA enrichment plot demonstrating the upregulation of genes involved in the pentose phosphate pathway in MB-R cells. **C** G6PD activity measured as the nmol of NADPH generated by the conversion of glucose-6-phosphate into 6-gluconic acid per minute. **D** Western Blot analysis of G6PD, TKT, and vinculin as loading control, in HD-MB03 and DAOY sensitive and resistant cells. **E** 6-phosphogluconic acid abundance normalized on proteins. **F** Dose-response analysis displaying the effect on cell viability of G6PDi in HD-MB03-S and HD-MB03-R cells as indicated. **G** Dose-response curves displaying the effect of the combination of G6PDi and VECC in HD-MB03-R and DAOY-R.

Considering that NADPH is the main electron donor for the regeneration of both GSH and Thioredoxin systems, we measured NADPH and NADP^+^ levels in MB cells. Although they both displayed a significant increase in HD-MB03-R cells, we observed a consistent increase in their NADPH/NADP^+^ ratio in MB-R cells (Fig. 2A, Fig. S1A). This suggests that MB-R cells enhance both NADP^+^ synthesis and NADPH regeneration to supply antioxidant systems and biosynthetic reactions. Gene set enrichment analysis (GSEA) performed on our previously published transcriptomic data [8] highlights the pentose phosphate pathway (PPP) as the most significantly enriched pathway within the KEGG gene set enrichment collection in MB-R cells (Fig. 2B, Fig. S1B). Since the oxidative branch of the PPP is considered the major source of NADPH inside the cells, by shunting glucose 6-phosphate (G6P) from glycolysis and thus generating NADPH and ribose 5-phosphate (R5P) [33], we hypothesized that MB-R cells might compensate their high NADPH demand by enhancing PPP activity. Based on these considerations, we measured the enzymatic activity of Glucose 6-Phosphate Dehydrogenase (G6PD), the rate-limiting enzyme of the oxidative branch of PPP (oxPPP), which displayed enhanced activity in MB-R cells relative to MB-S (Fig. 2C). Supporting these data, we observed a significant increase in both G6PD and Transketolase (TKT) expression levels in MB-R cells (Fig. 2D). Indeed, TKT is the rate-limiting enzyme of the non-oxidative branch of PPP, responsible for recycling metabolites back into glycolysis to replenish the oxidative phase, and further promoting NADPH synthesis [34]. As a further confirmation of the increased flux of glycolytic intermediates into the PPP, we measured the abundance of the key oxPPP downstream intermediate 6-phosphogluconate (6-PG). As depicted in Fig. 2E, HD-MB03-R cells are characterized by a significant accumulation of 6-PG. Moreover, stable isotope tracing using ^13^C-[U]-Glucose displayed a slight, although consistent, enrichment of ^13^C in d-Sedoheptulose-7-phosphate and d-Xylulose 5-phosphate in MB-R cells (Fig. S1C), further suggesting an increased metabolic flux through the PPP in resistant cells.

Interestingly, the cytotoxic effects of the pharmacological inhibition of G6PD using G6PDi-1 [35] were significantly greater in MB-R than MB-S cells (Fig. 2F–S1D) and further enhanced the effectiveness of chemotherapy when combined (Fig. 2G–S1E), indicating a partial dependence of MB-R cells on G6PD activity-dependent oxPPP fueling to guarantee the proper amount of reducing power through NADPH production.

### 3.3 Chemotherapy resistance is sustained by NRF2 activity

**Fig. 3:**
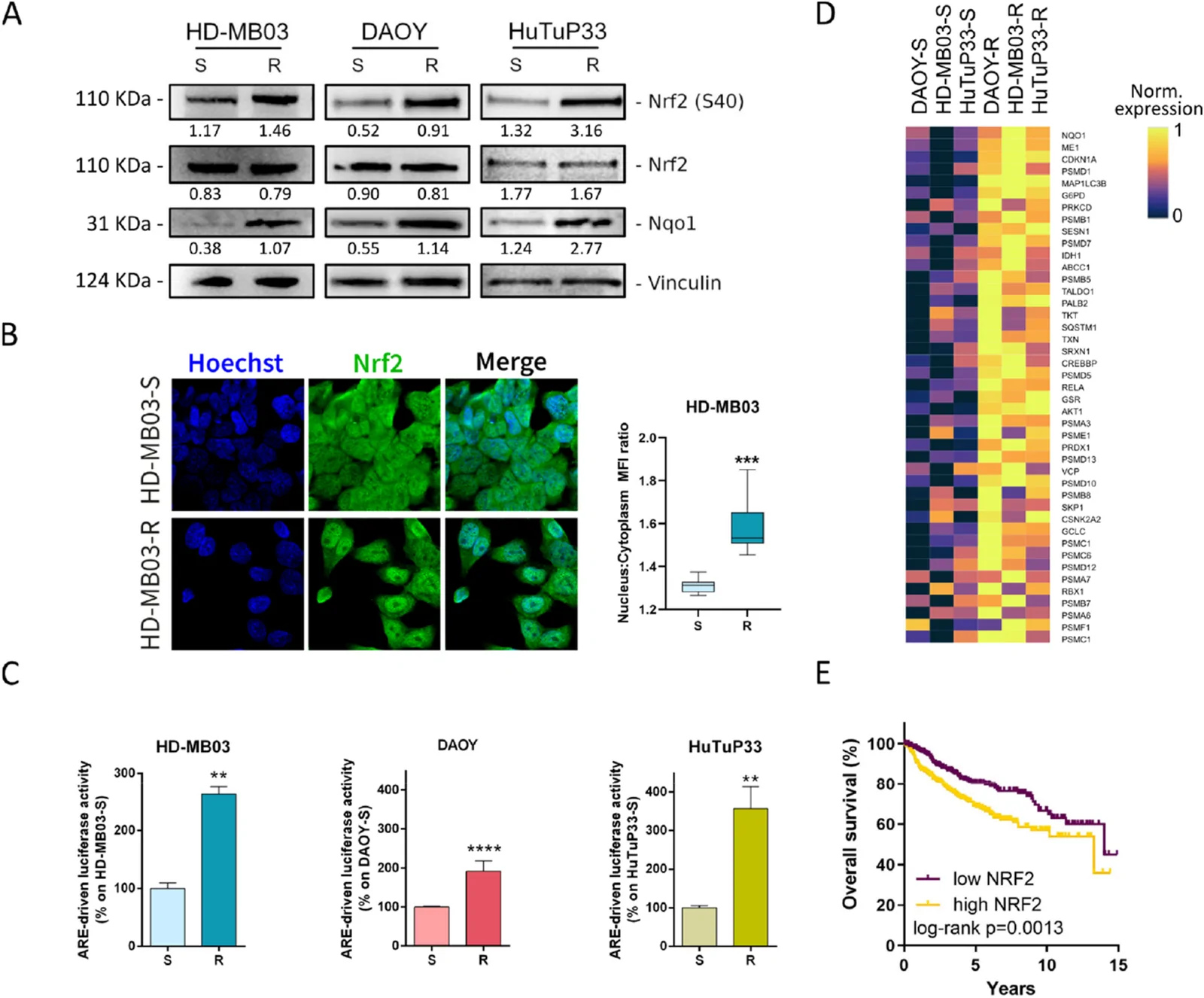
A Western Blot analysis of NRF2 (S40), NRF2, Nqo1, and vinculin as loading control, in HD-MB03, DAOY, and HutuP33 sensitive and resistant cells. **B** Immunostaining for NRF2 (green) in HD-MB03-S and HD-MB03-R cells. Nuclei were counterstained with Hoechst 33342 (blue). Box plots represent the ratio of MFI of green fluorescence (NRF2) between nucleus and cytoplasm of each cell. ***p < 0.001**C** NRF2 transcriptional activity was measured using the Antioxidant response element (ARE)-driven reporter assay in MB-S and MB-R cells as indicated. 24h after seeding, cells were transfected with PGL-8xARE plasmid (ARE-luc) together with pMAX-GFP plasmid. Luciferase activity and GFP fluorescence were detected 48 h after transfection. Data are expressed as a percentage of the relative light units, normalized to GFP signal, on MB-S cell relative cell lines. **p < 0.01, ***p < 0.001, ****p < 0.001 by multiple *t*-test comparison. **D** Heatmap displaying differentially expressed genes between MB-R and MB-S cells (p < 0.05, Holm–Šídák multiple *t*-test) belonging to REACTOME_KEAP1_NFE2L2_PATHWAY MSigDB gene set collection. Data are represented as the mean of 4 biological replicates. For visualization, expression values were normalized using min–max scaling for each gene, setting the minimum and maximum expression values to 0 and 1. Colors indicate relative expression levels. **E** Kaplan Meyer curves comparing the overall survival (OS) of MB patients (from GSE85217) with high and low NRF2 risk score calculated as described in the Materials and methods section.

Considering that redox homeostasis depends on a tightly regulated balance between ROS production and scavenging, and that cancer cells are able to modulate ROS levels to sustain chemotherapy resistance [36], we evaluated the expression and the activity of the NRF2 transcription factor, widely recognized as the master regulator of the antioxidant response in both normal and cancer cells [37]. Western blot analyses revealed increased levels of phosphorylated NRF2 (p-NRF2 (S40)), but not its total form (Fig. 3A, S2A), in MB-R cells. The impact of NRF2 phosphorylation at Ser40 on the activity of this transcription factor has been debated for years [38,39], however, within our model, the accumulation of p-NRF2 is clearly associated with a higher expression of its target gene NQO1, hence suggesting a phosphorylation-dependent enhanced NRF2 transcriptional activity (Fig. 3A). Indeed, resistant cells showed an increase in the phosphorylation of PKCα, the kinase responsible for NRF2 S40 phosphorylation (Fig. S2B). Moreover, direct inhibition of PKCα through the well-known specific inhibitor Enzastaurin, besides reducing its phosphorylation, decreased the levels of both p-NRF2(S40) and NRF2 target genes NQO1 and FTH1 (Fig. S2C and D). The overactivation of NRF2 signaling in MB-R was further evidenced by the augmented accumulation of NRF2 levels in the nucleus of MB-R (Fig. 3B–S2E,F), together with a significant increase of a luciferase-based reporter signal dependent on the NRF2 binding of the Antioxidant Response Element (ARE) consensus (Fig. 3C) [15]. Additionally, our gene expression data [8] confirm that VECC resistance is positively correlated with the upregulation of a series of genes associated with the activation of the Keap1/NRF2 pathway (Fig. 3D).

More interestingly, a NRF2-transcriptional activity-dependent risk score (based on the expression of NRF2 pathway associated genes reported in Fig. 3D and Table S1), was able to predict the survival of patients from a large pediatric MB expression cohort [7], with patients with a higher risk score displaying a worse prognosis (Fig. 3E). These data validate in a large cohort of MB-patients the relevance of Keap1/NRF2 axis in sustaining malignancy in MB tumors.

### 3.4 NRF2 signaling activation modulates ferroptosis resistance in chemotolerant cells

**Fig. 4:**
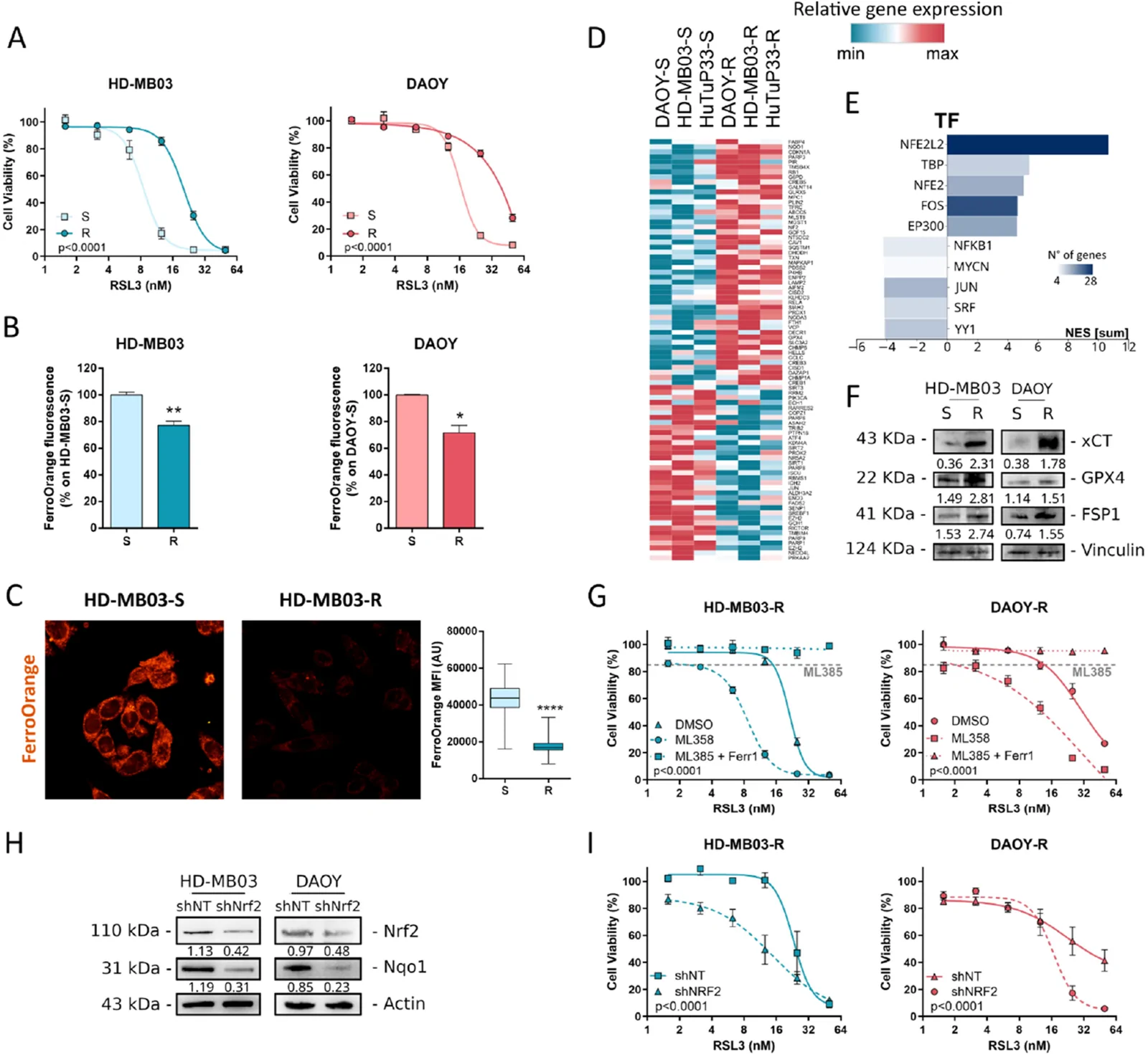
A Dose-response curves displaying increased resistance to RSL3 treatment (72h) of MB-R models relative to their sensitive counterparts (MB-S). Data are represented as the mean ± SEM of at least 3 replicates. **B** Flow-cytometry analysis of cellular labile iron pool, measured through the staining with FerroOrange probe, in MB-R and MB-S cells as indicated. Data are represented as the percentage of mean fluorescence intensity (MFI) of the dye over MB-S cells. *p < 0.05, **p < 0.01. **C** Fluorescence Microscopy analysis and relative quantification of labile iron pool, by staining cells with FerroOrange dye. Data are represented as the percentage of mean fluorescence intensity (MFI) of the dye ****p < 0.0001. **D** Heatmap displaying differentially expressed ferroptosis suppressor genes from FerroDb. Gene expression levels of FerroDb-listed genes were compared between MB-R and MB-S within each cell type. The relative expression of the genes that were consistently and significantly differentially expressed across all three cell lines (p < 0.01, Holm–Šídák multiple *t*-test) is shown in the heatmap. For visualization, the mean expression values across 4 biological replicates per cell line were independently normalized by min–max scaling for each gene, setting the minimum and maximum expression values to 0 and 1. Colors indicate relative expression levels. **E** Transcription factors regulating the differentially expressed genes displayed in panel D predicted by iRegulon analysis. **F** Western Blot analysis of xCT, GPX4, FSP1, and vinculin as loading control, in HD-MB03, DAOY sensitive and resistant cells. **G** Dose-response analysis of HD-MB03-R and DAOY-R cells treated with RSL3 alone and combined with the NRF2 inhibitor ML385 at the fixed concentration of 5 μM and or Ferrostatin (Ferr1) at the fixed concentration of 1 μM. Data are represented as the mean ± SEM of at least 3 replicates. **H** Western Blot analysis of NRF2 and Nqo1, and Actin as loading control, in HD-MB03 and DAOY transduced with a shNon Targeting (shNT) and shNRF2 vectors. **I** Dose-response analysis of HD-MB03-R and DAOY-R shNT and ShNRF2 cells treated with RSL3. Data are represented as the mean ± SEM of at least 3 replicates.

Several recent studies have highlighted cisplatin, cyclophosphamide, etoposide, and vincristine (composing the VECC cocktail) as inducers of ferroptosis, a programmed cell death mediated by oxidative stress and lipid peroxidation [14,40,41]. In addition, the involvement of the NRF2 pathway in controlling iron homeostasis and suppressing ferroptosis has been widely reported and associated with the modulation of antioxidant genes [28,29,42]. Therefore, we next investigated whether the acquisition of VECC resistance correlated with a concurrent resistance to ferroptosis induction. In this context, MB-R cells showed increased tolerance against GPX4 inhibition (through RSL3) or SLC7A11 blockade (through Erastin)-induced ferroptosis, relative to MB-S cells (Fig. 4A, Fig. S3A). Indeed, MB-R cells were characterized by reduced intracellular levels of labile iron(II) ions (Fe2^+^) (Fig. 4B and C), the fundamental mediators of ferroptosis initiation. Interestingly, the iron-sequestering protein and NRF2 target, FTH1 was found over-expressed in our MB-R cells (Fig. S3B), thus providing a potential explanation for the observed restriction of free iron levels and the consequent increased resistance to ferroptosis, as already suggested [43].

To gain a comprehensive understanding of this phenomenon and even identify additional mediators of ferroptosis resistance in MB-R cells, we evaluated, in our models, the expression of a series of ferroptosis suppressors described in the dedicated database FerrDb V2 [44], by reanalyzing our previously published gene expression dataset (GSE220543) [8]. The heatmap in Fig. 4D distinctly shows 85 significantly deregulated genes, recognized as suppressors of ferroptosis, in MB-R cells (Table S3). Since ferroptosis could be mediated and controlled by different transcription factors, including NRF2, Bach1 [28], NUPR1 [45], TP53, and HIF-1α [46], we constructed a transcriptional regulatory network for the significantly differentially expressed ferroptosis suppressor genes using the iRegulon tool to enrich transcription factor binding motifs [47]. The analysis suggests that NRF2 may serve as the primary transcriptional regulator of the overexpressed genes. However, no specific transcription factor was identified as a likely regulator of the downregulated genes (Fig. 4E). Supporting this hypothesis, the protein levels of the ferroptosis suppressors GPX4, xCT, and Ferroptosis Suppressor Protein 1 (FSP1), all well-described NRF2 target genes [48,49], were found to be overexpressed in MB-R cells (Fig. 4F).

To definitively validate the potential role of NRF2 in sustaining ferroptosis resistance in MB-R cells, we suppressed its transcriptional activity by using the specific inhibitor ML385 [50]. ML385 strongly sensitized MB-R cells to both RSL3 and Erastin, confirming that NRF2 plays a crucial role in supporting ferroptosis resistance in chemotherapy-resistant cells (Fig. 4G and Fig. S3C). Similar effects were observed in MB-R cells after NRF2 knockdown (Fig. 4H), confirming once again that the activation of NRF2 associated with chemotherapy resistance protects MB-R cells also against ferroptosis induction.

### 3.5 NRF2 sustains detoxification of ROS in MB-resistant cells

Our data converge to the hypothesis that the enhancement of NRF2-driven antioxidant defense systems may be involved in the development of chemotherapy resistance in MB.

**Fig. 5:**
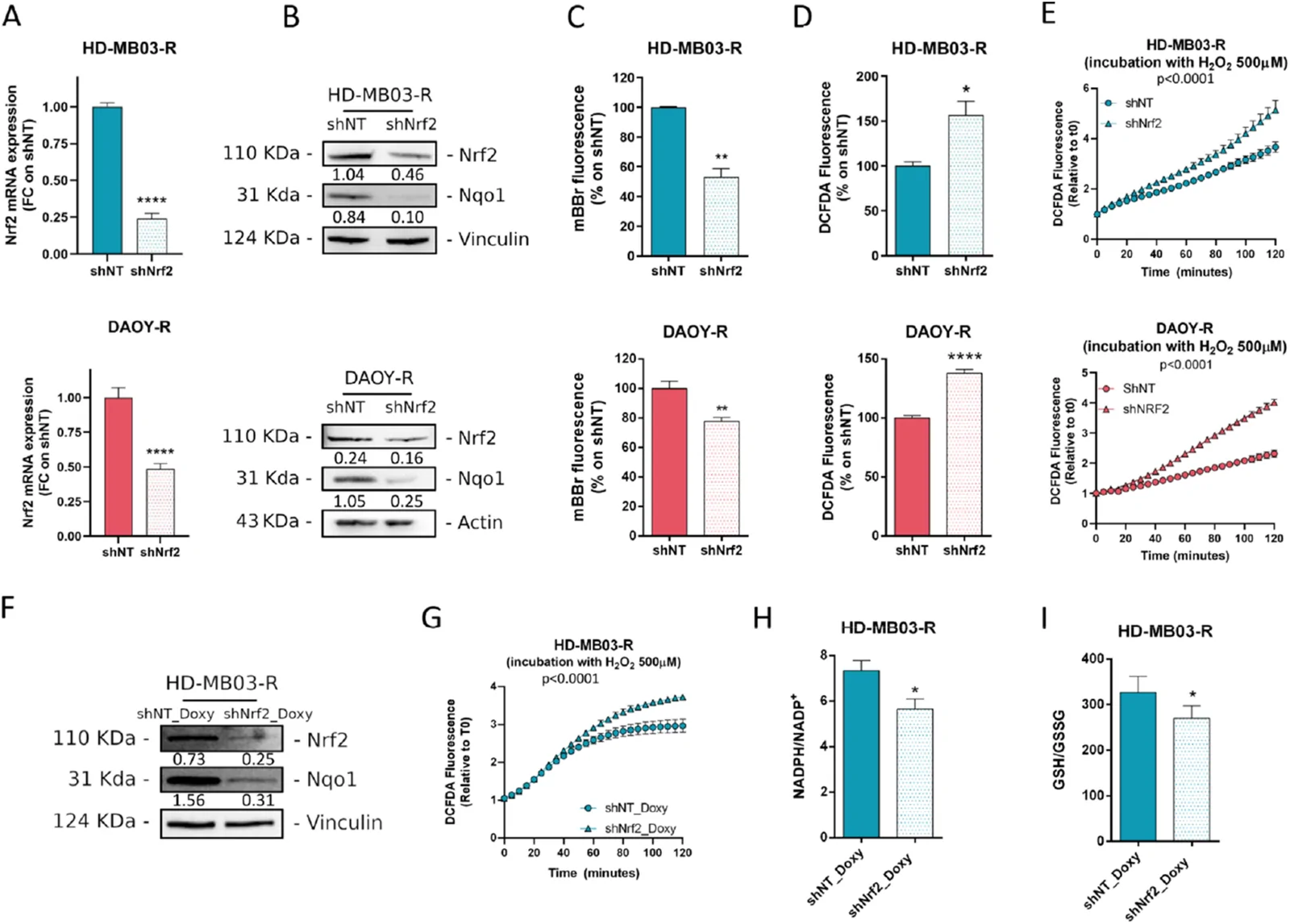
A mRNA expression levels of NRF2 after NRF2 knockdown. Data are represented as fold change over MB-R cells transduced with a non-targeting control vector. mRNA expression values were normalized to GUSB according to the ΔΔCt method. ****p < 0.0001. **B** Western Blot analysis of NRF2 and Nqo1, and Vinculin or Actin as loading control, in MB-R cells transduced with a shNon Targeting (shNT) and shNRF2 vectors. **C** Flow cytometry analysis of GSH content in NRF2 KD cells using MBB staining. Data are represented as a percentage relative to the cells transduced with the control vector. **D** Flow cytometry analysis of ROS content in NRF2 KD cells by DCFDA staining. Data are represented as a percentage relative to the cells transduced with the control vector. **E** ROS levels during oxidative stress induced by the incubation with hydrogen peroxide (500 μM) were measured over time (120 min) in HD-MB03-ShNT/ShNRF2 and DAOY-ShNT/ShNRF2 cells. Following the staining with DCFDA probe, hydrogen peroxide was added, and the fluorescence of the oxidized dye was recorded from time 0 every 5 min. **F** Western Blot analysis of NRF2 and Nqo1, and vinculin as loading control, in HD-MB03 transduced with a shNT and shNRF2 doxycyclin-inducible vectors, upon doxycycline treatment. **G** ROS levels during oxidative stress by incubation with hydrogen peroxide (500 μM) were measured over time (120 min) in HD-MB03 transduced with shNT and shNRF2 inducible vectors, upon doxycycline treatment. Following the staining with DCFDA probe, hydrogen peroxide was added, and the fluorescence of the oxidized dye was recorded from time 0 every 5 min **H** NADPH/NADP ^+^ ratio and **I** GSH/GSSG ratio measured by LC-MS/MS in HD-MB03 transduced with a shNT and shNRF2 inducible vectors, upon doxycycline treatment. *p < 0.05; **p < 0.01; ****p < 0.0001 vs. shNT cells.

To further evaluate the contribution of NRF2 signaling in sustaining the antioxidant program of resistant cells, we measured free thiol content and ROS levels in NRF2-silenced MB-R cells (Fig. 5A and B, S4A). NRF2 silencing induced a significant reduction of reactive-thiol content (Fig. 5C–S4B) and a prominent increase of basal ROS levels (Fig. 5C). Moreover, to functionally validate these results, we measured ROS accumulation over time upon induction of oxidative stress through H_2_O_2_. Interestingly, NRF2 suppression reduced MB-R ability to scavenge ROS, allowing their significant accumulation relative to NRF2-competent cells (Fig. 5E). As further confirmation, the specificity of NRF2 knockdown-dependent effects was validated in HD-MB03-R conditionally silenced for NRF2 through doxycycline treatment (Fig. 5F and G). More importantly, LC-MS analysis revealed that NRF2 suppression affects both NADPH/NADP^+^ and GSH/GSSG ratios (Fig. 5H and I), confirming that reduction of NRF2 levels limit the availability of molecules with high reducing power and providing further evidence that NRF2 plays a crucial role in orchestrating intracellular antioxidant mechanisms in chemoresistant MB cells.

### 3.6 NRF2 sustains chemotherapy resistance by controlling the antioxidant response in chemoresistant MB cells

Results collected so far highlight that chemotherapy-resistant MB cells are characterized by improved antioxidant defense, with NRF2 activation potentially acting as a primary mediator of these effects.

**Fig. 6:**
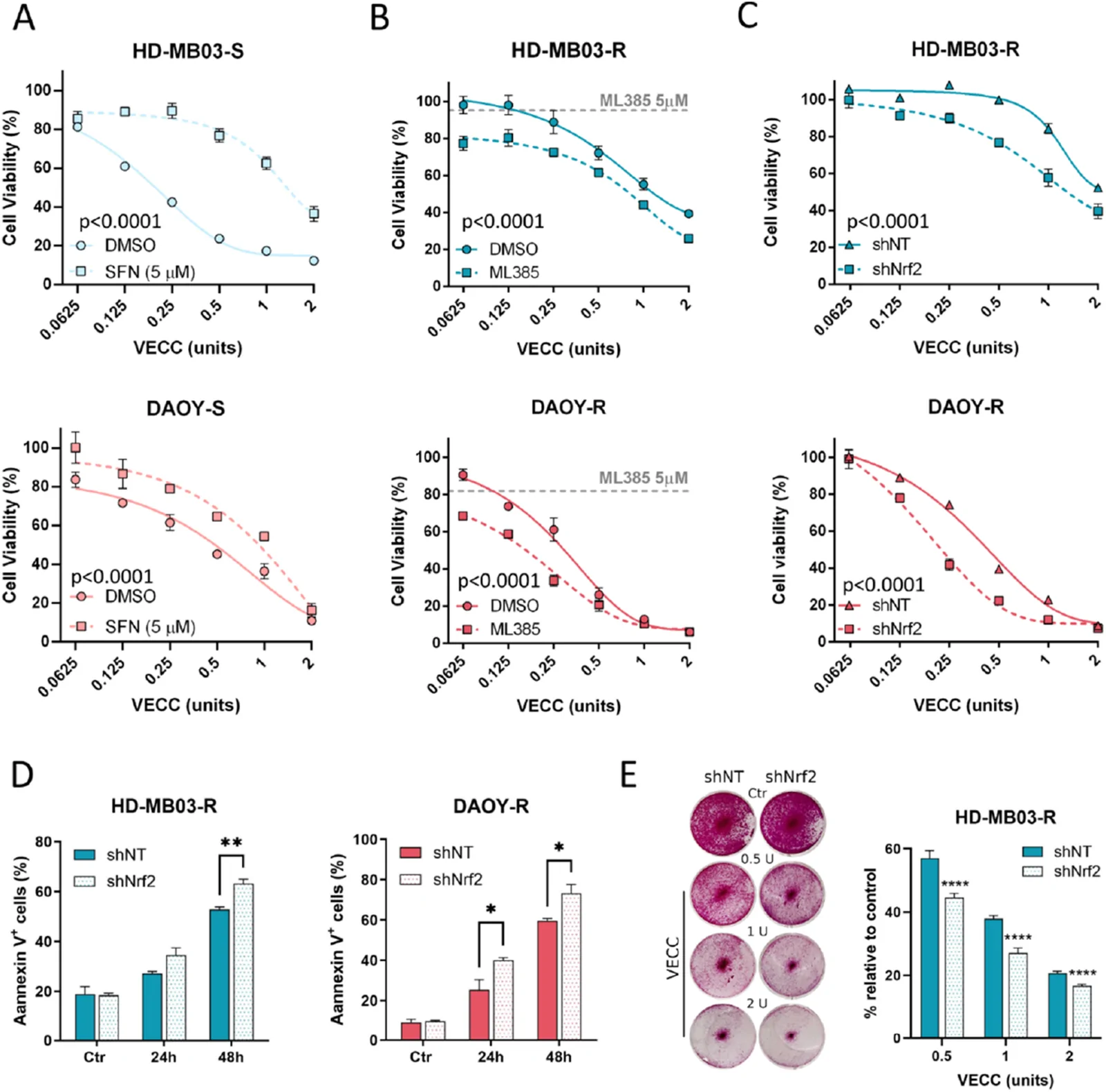
A Dose-response analysis showing the effect of VECC treatment in combination with the NRF2 activator sulforaphane (SFN) on MB-S cell viability. **B** Dose-response analysis displaying the effect of VECC in combination with NRF2 inhibitor ML385 on MB-R cell viability. **C** VECC effects on cell viability of MB-R cells with NRF2 knockdown (shNRF2) or transduced with a control vector (shNT). **D** Bar plot showing the % apoptotic cells (Annexin V positive cells) after incubation of MB-R transduced with shNT or shNRF2 with 1 unit of VECC. Data are represented as the mean ± SEM of 3 independent experiments. *p < 0.05, **p < 0.01**E** Representative images of sulphorhodamine staining to evaluate the viability of MB-R cells transduced with shNRF2 or the control vector after 72h of VECC treatment at the indicated doses and the relative quantification expressed as % on untreated cells. Data are represented as the mean ± SEM. ****p < 0.0001.

To assess the direct and functional role of NRF2 activity in supporting chemoresistance of MB cells, we evaluated whether overactivation of NRF2 would play a role in the acquisition of resistant traits in MB cells. To this aim, we tested the sensitivity of MB-S cells to VECC upon stimulation with the NRF2 activator sulforaphane (SFN) [51]. Importantly, activation of NRF2 strongly protected all the MB-S cells tested from chemotherapy. (Fig. 6A). On the contrary, MB-R cells in which NRF2 activity had been specifically inhibited through ML385 or genetically suppressed all displayed a significant increased sensitivity to VECC (Fig. 6 B,C, S5A,B). Interestingly, similar effects were observed by inhibiting NRF2 phosphorylation (Ser40) with the PKC inhibitor Enzastaurin (Fig. S5C and D). These data were further validated by showing increased apoptotic cell death (VECC-induced) in NRF2-silenced MB-R cells (Fig. 6D). Finally, Sulforhodamine B (SRB) staining definitely remarked an increased effect of VECC treatment in NRF2-silenced HD-MB03-R cells (Fig. 6E).

**Fig. 7:**
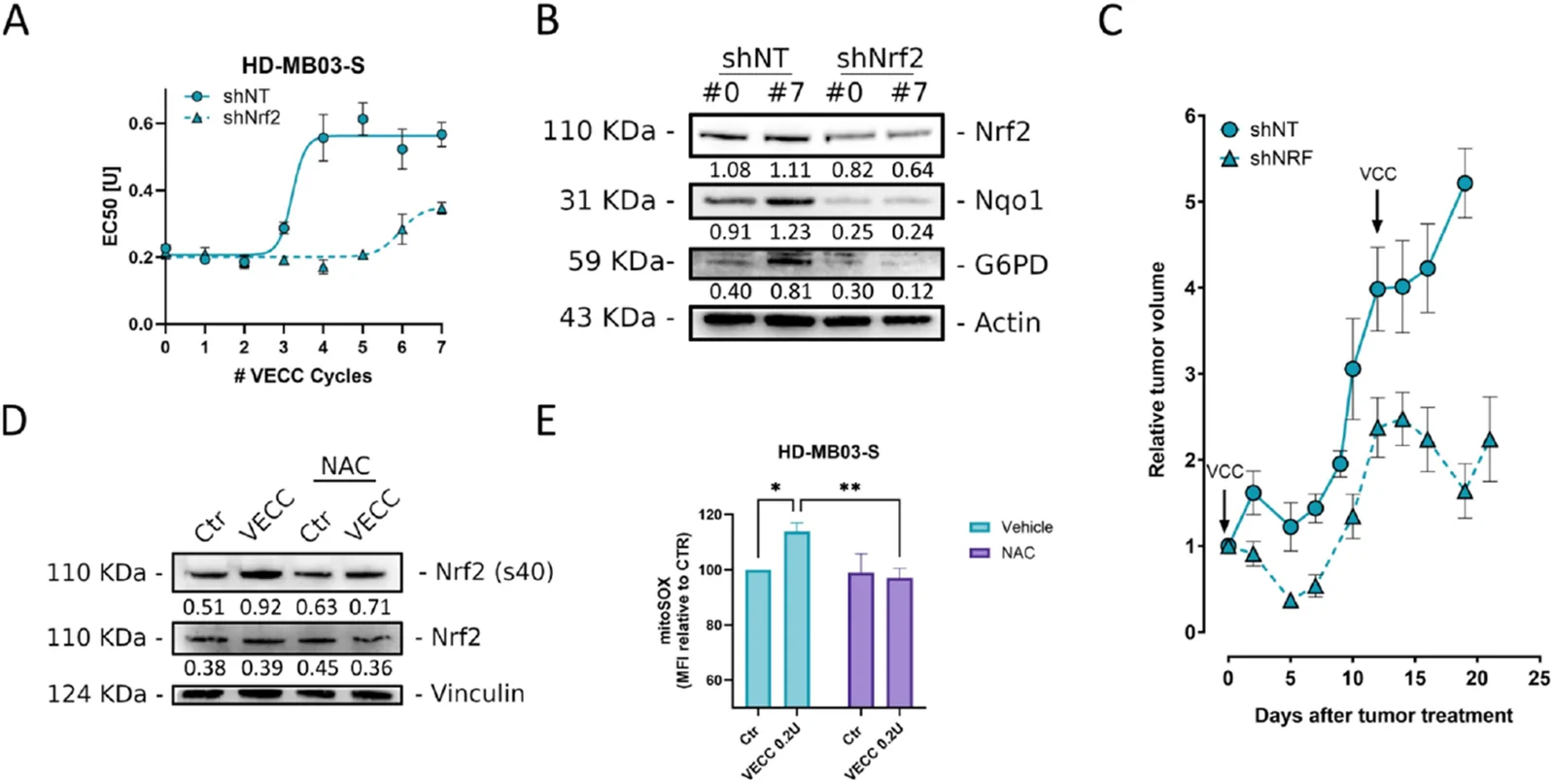
A Acquisition of chemoresistance in HD-MB03-S shNT and shNRF2 cell lines is shown by plotting EC50 of every weekly VECC cycle of treatment. EC50 was evaluated after 72h of VECC exposure. **B** Immunoblot analysis and quantification, relative to b-actin expression, of NRF2 and its target genes Nqo1 and G6PD in HD-MB03 stably expressing shNT or shNRF2 before (#0) and after the exposure to 7 cycles of VECC (#7) **C** Changes in tumor burden in a xenograft model of chemotherapy resistance acquisition using HD-MB03-shNT and HD-MB03-shNRF2 cells. When tumors reached a volume of 50 mm^3^, mice received the first cycle of vincristine, cisplatin, and cyclophosphamide (VCC). After a 12-day recovery period, animals were administered a second treatment cycle. **D** Immunoblot analysis and quantification of Nrf2 and phospho-Nrf2 (Ser40) expression, normalized to Vinculin, in HD-MB03-S cells treated with VECC (0.2 U for 24 h) alone or in the presence of NAC (1 mM). **E** Following 24 h of treatment with VECC (0.2 U) alone or in the presence of NAC (1 mM), the mtROS were evaluated in HD-MB03-S cells by flow cytometry. Data are represented as the mean ± SEM. *p < 0.05 **p < 0.01.

Our chemotherapy-resistant MB models were generated by weekly exposing cells to sublethal doses of VECC [8]. Considering that adaptive chemotherapy resistance could be overcome by a temporally controlled intervention against vulnerable nodes [52], to confirm the role of NRF2 transcriptional activity in sustaining adaptation to chemotherapy and acquisition of chemoresistant traits, we evaluated whether NRF2 knockdown would be sufficient to prevent the occurrence of chemoresistance in MB-S cells. As expected, control HD-MB03-S cells rapidly acquired resistance to the chemotherapeutics, displaying an increase in VECC IC_50_ starting from the 3rd cycle of VECC exposure. Intriguingly, NRF2-silenced HD-MB03-S cells were not able to efficiently adapt to chemotherapy, showing a slight reduction in VECC sensitivity only at later time points, starting from cycle 6 (Fig. 7A). Western blot analysis confirmed NRF2 transcriptional activation in the newly obtained shNT-HD-MB03-R cells (#7), with an increase in the expression of the NRF2 targets NQO1 and G6PD. On the contrary, the expression of both proteins was maintained low throughout VECC cycles in NRF2-silenced HD-MB03 when analyzed at the same VECC cycle (Fig. 7B). This data demonstrated for the first time that NRF2 is directly required for the acquisition of chemotherapy resistance in MB cells. As a further evidence of the pivotal role played by NRF2 in orchestrating the response to chemotherapy and the acquisition of chemotherapy resistance in MB, we performed in vivo experiments in which we monitored the progression of shNT and shNRF2-HD-MB03-S tumors following two cycles of chemotherapy with vincristine, cyclophosphamide, and cisplatin (VCC). As shown in Fig. 7C, shNRF2 tumors displayed a better response to the first cycle of VCC, with a clear early reduction of tumor size that was not observed in shNT tumors. Despite shNRF2 tumors resumed growth within 5 days from treatment, the second VCC cycle exhibited a consistent shrinkage of tumor size and growth arrest. Importantly, control shNT HD-MB03 tumors, which showed a partial response after the first VCC cycle, displayed total insensitivity to the second VCC exposure, with no sign of tumor regression or proliferative arrest (Fig. 7C).

Overall, these findings are consistent with our *in vitro* observations and suggest that NRF2 activity can contribute to the development of a chemotherapy-resistant phenotype in MB cells.

It is well known that many chemotherapeutic agents increase intracellular ROS levels, and their anticancer effects are largely attributed to the induction of oxidative stress and ROS-driven cell damage. Considering that mild mitochondrial oxidative stress can lead to persistent activation of cytoprotective processes, within the so-called mitohormesis phenomenon, we wondered whether NRF2, as the master regulator of the intracellular redox status, might be involved in these adaptive responses [53]. To explore this possibility, we treated MB-S cells with sublethal VECC concentrations (the same used to generate MB-R cells) in combination with the well-known antioxidant N-acetylcysteine (NAC). We observed that VECC treatment alone induced a mild increase in mitochondrial ROS (mtROS) levels accompanied by a concomitant elevation of phosphorylated NRF2 (p-NRF2). Notably, co-treatment with NAC restored mtROS levels to a basal condition and limited NRF2 phosphorylation when compared with VECC alone. These results support our hypothesis that mild chemotherapeutic stress promotes mtROS generation, which in turn activates the NRF2 signaling pathway.

## 4 Discussion

Despite recent progress in the clinical management of MB, approximately 30% of patients experience tumor relapse with metastatic recurrent disease that is often refractory to further chemotherapy treatments. Our previous work suggests that the functional evolution of MB cells in response to therapies may occur through the adaptation of intracellular metabolic activities eventually aimed at supporting the biosynthetic demands of increased malignancy and the oxidant detoxification processes [8,54]. Chemotherapeutic agents commonly included in MB therapeutic protocols, in part, exert their anti-tumor effects by inducing oxidative stress. Indeed, the adaptation of cancer cells needed to counteract this stress, through the maintenance of a tightly regulated redox balance, is emerging as a hallmark of cancer resistance [55,56].

In this context, our results indicate that, upon exposure to several cycles of chemotherapy, MB-R cells develop a stable adaptation to oxidative stress by acquiring enhanced antioxidant capacities through increased activity of two major thiol-dependent antioxidant systems: the glutathione and thioredoxin systems, which have already been identified as relevant for cancer initiation and progression [32]. As a consequence, resistant cells are characterized by reduced basal levels of ROS and an enhanced ability to buffer them even upon oxidative insults.

We focused our attention on the metabolic processes that regenerate NADPH, finding that MB-R cells exhibit an increased NADPH/NADP^+^ ratio, indicating a higher reservoir of reducing power that chemotolerant cells can exploit. Although NADPH is produced and consumed by several metabolic reactions, the oxidative branch of the pentose phosphate pathway (PPP) is considered its primary source, specifically through the activity of G6PD (glucose-6-phosphate dehydrogenase), which metabolizes glucose-6-phosphate into 6-phosphogluconate and generates one molecule of NADPH from NADP^+^ [33]. Our data show that MB-R cells possess increased G6PD expression and activity, as shown by over-accumulation of 6-phosphogluconate. Besides NADPH generation for ROS protection, PPP is exploited by cancer cells to produce high levels of nucleotides for DNA synthesis and repair [57], in line with previously reported results of our group that highlight nucleoside metabolism as a relevant vulnerability in chemoresistant MB models [4,8]. These findings strongly support the hypothesis that MB-R cells increase the flux of glucose through the PPP to meet the demand for NADPH, corroborating findings from other cancer models where PPP activation confers a survival advantage of cells by maintaining their redox balance ​ [57].

The regulation of ROS scavenging systems and redox homeostasis in cells is tightly controlled at the transcriptional level, with NRF2 acting as their master regulator by driving the expression of several proteins involved in the metabolism of GSH and NADPH and by directly regulating G6PD, NQO1, and other enzymes that ensure protection against oxidative stress [17,21]. This study provides compelling evidence that NRF2 signaling plays a pivotal role in mediating chemotherapy and ferroptosis resistance in MB cells. We demonstrate that NRF2-dependent redox homeostasis is a key adaptive mechanism that enables resistant MB cells to thrive under chemotherapy-induced oxidative stress. These findings build on previous reports that highlight the role of NRF2 in cancer progression and treatment resistance, underscoring its function as a double-edged sword in cancer biology. Tight regulation of the redox homeostasis through NRF2 activity not only sustains the survival of normal cells but also fosters specific conditions that enhance the proliferation and persistence of cancer cells [10,23,58]. In this context, many reports have demonstrated the contribution of NRF2 in protecting ovarian cancer cells from ROS-mediated damage induced by several agents, including cisplatin, paclitaxel, PARP inhibitors, pertuzumab, and trastuzumab [59]. Moreover, high levels of NRF2 have been found in higher-risk patients affected by myelodysplastic syndromes, here sustaining cytarabine resistance partially through the activation of its direct target gene DUSP1 [60]. Finally, genomic alterations of *KEAP1* and *NFE2L2* that lead to NRF2 stabilization are frequent events in patients with lung adenocarcinoma and with lung squamous cell carcinoma and have an impact on treatment response [61].

Our investigation reveals that the acquisition of chemotherapy resistance in MB cells is accompanied by the transcriptional activation of NRF2, through increased levels of NRF2 phosphorylation (S40) rather than a general increase in its protein expression. The phosphorylation of NRF2 at Ser40 by protein kinase C (PKC) has been proposed to facilitate the release of NRF2 from KEAP1 and promote its nuclear translocation. However, this mechanism remains controversial, as conflicting studies have challenged the need of this phosphorylation site for NRF2 activation. Huang et al. [38] suggested that Ser40 phosphorylation enhances NRF2 translocation and subsequent ARE-dependent transcriptional activity. Conversely, Bloom and Jaiswal [39] argued that Ser40 phosphorylation may not be essential for NRF2 stabilization and nuclear accumulation. In our study, increased levels of phosphorylated NRF2 (S40) are associated with enhanced transcriptional activity and upregulation of target genes such as NQO1. In addition, direct inhibition of PKC using the well-established PKC inhibitor Enzastaurin reduces pNRF2(Ser40) levels along with its target gene NQO1. Altogether, these results suggest that Ser40 phosphorylation should play a functional role in NRF2-driven chemoresistance in MB. Nevertheless, our data do not exclude the possibility of alternative or compensatory mechanisms contributing to NRF2 activation in chemotolerant cells.

The transactivation of NRF2 in MB-R cells and the upregulation of its target genes involved in antioxidant defense are consistent with other studies showing that NRF2 can be induced by chemotherapy as a protective response, enabling cancer cells to counteract drug-induced oxidative damage​ [15,16,24,61,62]. Additionally, Kaplan-Meier analysis of pediatric MB patient data revealed a significant correlation between high NRF2 activity and poor overall survival, suggesting that increased NRF2 activation could serve as a good prognostic indicator of the aggressive and even chemoresistant MB subtypes.

Another relevant aspect of our study is the correlation between chemotherapy resistance and resistance to ferroptosis induction. Oxidative stress and low intracellular antioxidant systems are the main causes of iron-dependent lipid peroxidation during ferroptosis. Considering that NRF2 target genes play a critical role in mediating iron/metal metabolism, intermediate metabolism, and glutathione synthesis/metabolism, we wondered if the activation of NRF2 in chemotherapy-resistant MB cells was also correlated with resistance to ferroptosis. Our results show that MB-resistant cells exhibit increased tolerance against ferroptosis inducers such as RSL3 and Erastin. This resistance is associated with lower levels of labile iron pool and higher expression of ferroptosis-suppressing genes under the direct transcriptional control of NRF2. These findings support previous evidence that NRF2 activation can protect cells from ferroptosis by upregulating iron sequestration proteins and antioxidant defenses, such as GPX4, which mitigate lipid peroxidation [63]. More importantly, these results highlight that chemotherapy-induced adaptive mechanisms could protect cancer cells from the insult provided, not only by chemotherapy itself, but also by other oxidative agents.

NRF2 knockdown in resistant cells impairs the ability of resistant cells to counteract oxidative stress and sensitizes cells to chemotherapy. These data are confirmed by NRF2 pharmacological inhibition with ML385, suggesting that NRF2 inhibition could be exploited to overcome resistance and improve treatment efficacy. Additionally, given that NRF2 knockdown enhanced ferroptosis sensitivity in our resistant cell models, the combination of NRF2 inhibitors with ferroptosis inducers may represent a promising therapeutic approach. However, although NRF2 inhibition might be considered therapeutically relevant, the broader impact of its inhibition in normal cells must be carefully evaluated, as NRF2 plays a crucial role in protecting healthy tissues from oxidative damage. On the other hand, exploring the combination of NRF2 inhibitors with other metabolic or redox modulators could provide new avenues for overcoming resistance in MB and potentially other aggressive cancers characterized by high NRF2 activity.

In addition to the identification of NRF2 as a critical driver of therapy resistance in MB-R cells, this work provides the first evidence that NRF2 represents a *conditio* sine *qua non* for the acquisition of chemotherapy resistance in MB. Our hypothesis, supported by the observation that NRF2 knockdown prevents MB-S cells from acquiring resistance within the expected VECC cycles, is that chemotherapy-induced ROS spikes could trigger specific adaptive processes such as hormesis or more specifically mitohormesis [53]. Indeed, a modest, although recurring increase in ROS and mtROS, dependent on a direct or even indirect chemotherapy-induced activity, could activate a stable adaptation towards high ROS levels. Our data suggest that this process may be relevant for the acquisition of chemotherapy resistance in MB cells, as VECC treatment induces a mild increase in mitochondrial ROS and NRF2 phosphorylation, both effects reversed by NAC. We suggest that this adaptation could be mediated by NRF2, whose activation enables cells to better manage oxidative stress through the transcriptional activation of genes involved in ROS detoxification, redox homeostasis, and mitochondrial biogenesis. Consistent with our *in vitro* studies, in vivo mouse xenograft models support the hypothesis that a proper functionality of the NRF2 pathway may contributes to the onset of chemotherapy resistance in medulloblastoma.

## 5 Conclusions

In conclusion, our findings underscore the relevance of the redox homeostasis controlled by NRF2 in sustaining chemotherapy and ferroptosis resistance in medulloblastoma. The activation of NRF2 confers a survival advantage to cells by enhancing the antioxidant defenses and mitigating oxidative stress, highlighting its potential as a target for overcoming therapy resistance. Developing strategies to inhibit NRF2 signaling could offer a promising therapeutic approach to enhance the effectiveness of current treatment regimens for medulloblastoma.

## CRediT authorship contribution statement

**Lorenzo Manfreda:** Conceptualization, Investigation, Visualization, Writing – original draft, Methodology, Data curation, Formal analysis. **Fatlum Rruga:** Conceptualization, Investigation, Methodology. **Arianna Zanolli:** Investigation, Methodology. **Bryan P. Marzullo:** Investigation. **Lisa Vettore:** Conceptualization. **Martina Mozzo:** Investigation. **Martina Canton:** Validation. **Chiara Marchioro:** Validation. **Elena Rampazzo:** Methodology, Writing – review & editing. **Elena Mariotto:** Writing – review & editing. **Serena Filiberti:** Investigation, Methodology, Validation. **Marta Turati:** Resources. **Roberto Ronca:** Supervision, Methodology. **Daniel A. Tennant:** Writing – review & editing, Supervision. **Luca Persano:** Writing – review & editing, Funding acquisition, Formal analysis. **Giampietro Viola:** Funding acquisition, Writing – review & editing. **Roberta Bortolozzi:** Conceptualization, Investigation, Project administration, Formal analysis, Supervision, Visualization, Writing – original draft, Methodology.

## Funding statement

This work was supported by the Rally Foundation for Childhood Cancer Research (#20IN28 and #21IC16 to LP), Cassa di Risparmio di Padova e Rovigo (CARIPARO) Foundation (Project No. 20/16 FCR to LP), Fondazione Just Italia (“Piu forti di tutto” to GV), and Associazione Italiana per la Ricerca sul Cancro (AIRC) (IG #23109 to GV). ER was supported by a fellowship from the Umberto Veronesi Foundation (#3628). EM was supported by a fellowship from AIRC (ID 24185). LV was supported through a grant from Cancer Research UK (C42109/A26982). LM was supported by the Società Italiana di Farmacologia (SIF research grants for short periods abroad) and by the EACR (#849). RR was supported by AIRC (IG #23151) and Associazione Dedicato A Te. MC was supported by Fondazione Città della Speranza PhD scholarship. SF was supported by Consorzio Interuniversitario per le Biotecnologie (CIB). We would like to acknowledge the support and resources of the Birmingham Metabolic Tracer Analysis Core.

## Declaration of competing interest

The authors declare that they have no known competing financial interests or personal relationships that could have appeared to influence the work reported in this paper.
