# Supplementary material for: NRF2 activation is required for chemotherapy resistance acquisition in medulloblastoma via metabolic and redox adaptation
Source: Redox Biol. 2026 Sep 3;97:104380. doi: 10.1016/j.redox.2026.104380 (PMC13579287; doi:10.1016/j.redox.2026.104380)
Supplement: Supplementary file 1 [file mmc1.pdf]

Supplementary Information for

NRF2 activation is required for chemotherapy resistance acquisition in medulloblastoma via metabolic and redox adaptation

Lorenzo Manfreda*, Fatlum Rruga, Arianna Zanolli, Bryan Marzullo, Lisa Vettore, Martina Mozzo, Martina Canton, Chiara Marchioro, Elena Rampazzo, Elena Mariotto, Serena Filiberti, Marta Turati, Roberto Ronca, Daniel A Tennant, Luca Persano, Giampietro Viola, Roberta Bortolozzi*

*Corresponding author. Email: roberta.bortolozzi@unipd.it; lorenzo.manfreda@unimib.it

This PDF file includes

supplementary figures S1 to S5

Other Supplementary Information for this manuscript includes:

Supplementary tables S1 to S3

Supplementary figure S1

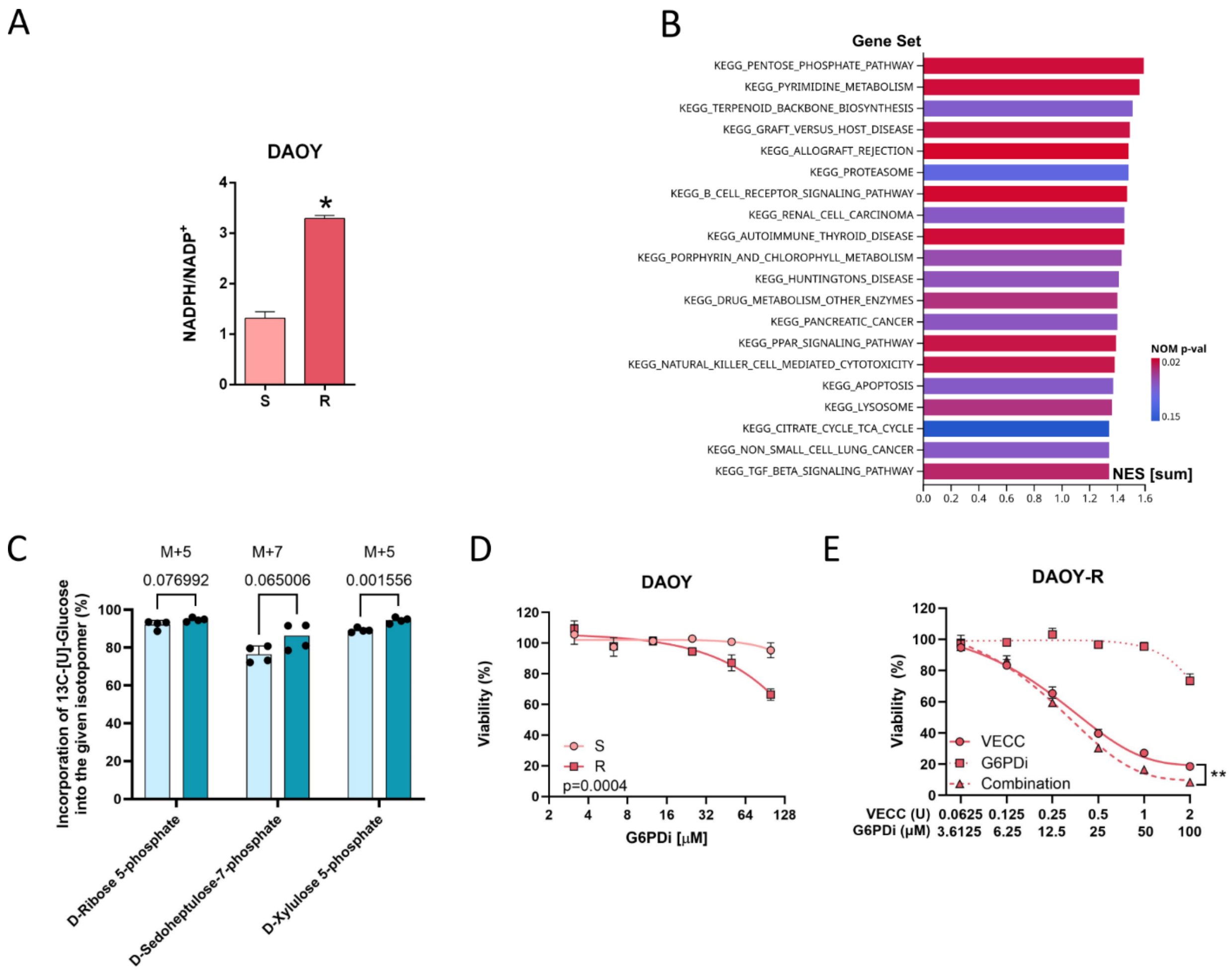

Fig. S1 A NADPH/NADP+ content ratio determined by enzymatic analysis of cell extract in DAOY-S and DAOY-R cells, as indicated. **B** Geneset enrichment analysis of the MB-R vs MB-S transcriptome, using the KEGG pathway database. **C** Percentage of the incorporation of 13C-[U]-Glucose in the indicated isotopomers **D** Dose-response analysis displaying the effect on cell viability of G6PDi in DAOY-S and DAOY-R cells as indicated. **E** Dose-response curves displaying the effect of the combination of G6PDi and VECC in DAOY-R.

Supplementary figure S2

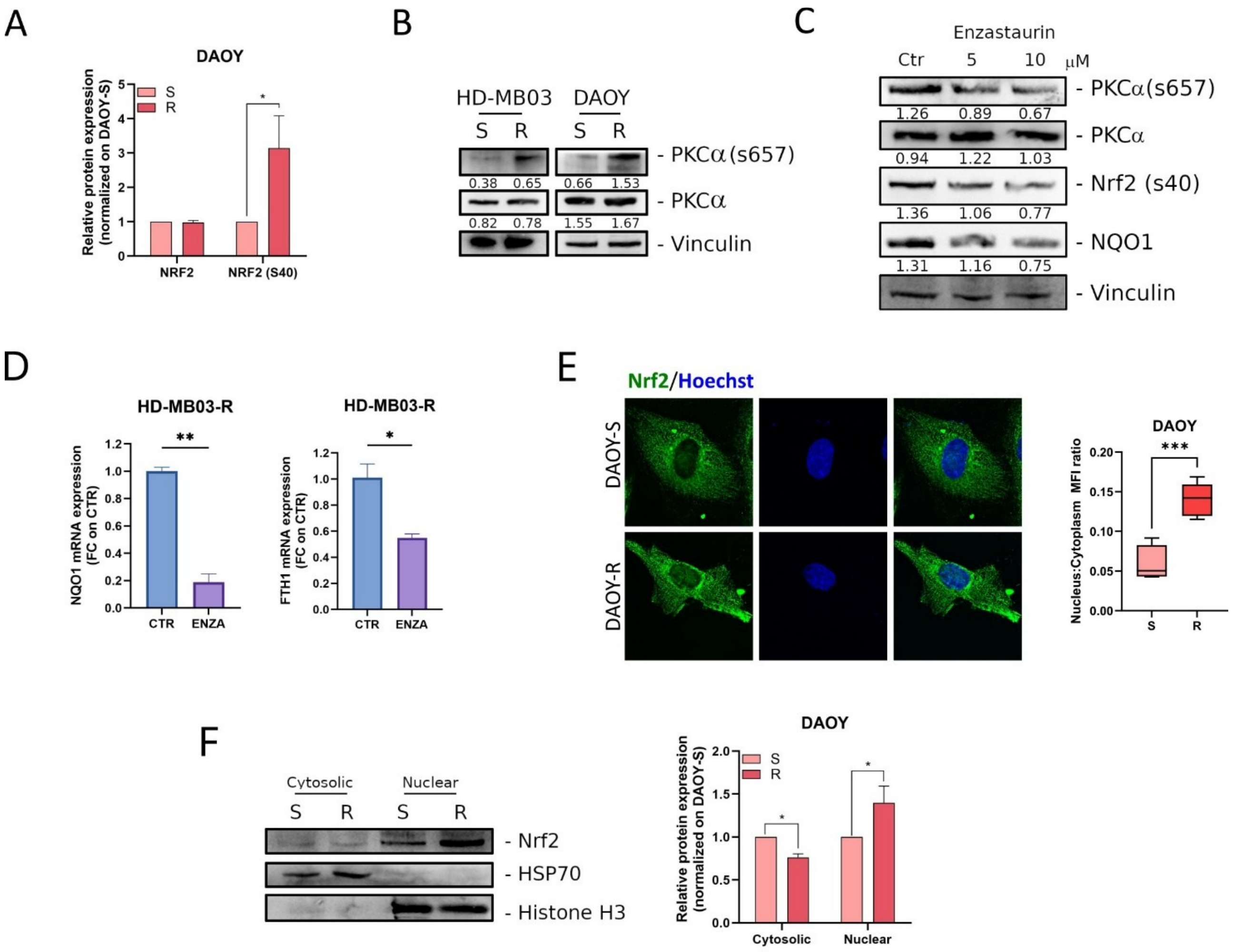

Fig S2 A Quantification of Nrf2 and Nrf2 (s40) protein levels based on densitometric analysis of the western blot shown in Fig3A, normalized to vinculin. Data are expressed as the mean±SEM from 3 independent experiments. $*P < 0.05$ compared to DAOY-S. **B-C** Western blot analysis on HD-MB03-R cells displaying **B** the expression of the phosphorylated PCKα (s657) in MB-S and MB-R cells and **C** the effect of 24 h of treatment with enzastaurin on PCKα (s657) and Nrf2 (s40) phosphorylation and Nqo1 expression, in HD-MB03-R cells. Total PKCα and Vinculin have been included as loading controls. **D** Effects of enzastaurin on NQO1 and FTH1 mRNA expression. HD-MB03-R cells were treated with enzastaurin 10 μM for 24 h; data are expressed as the mean ±SEM of the fold change relative to control. mRNA expression values were normalized to GUSB according to the ΔΔCt method. *p<0.05; **p<0.01 **E** Immunostaining for NRF2 (green) in DAOY-S and DAOY-R cells. Nuclei were counterstained with Hoechst 33342 (blue). (NRF2) normalized on blue fluorescence (Hoechst 33342). Box plots represent the ratio of MFI of green fluorescence (Nrf2) between the nucleus and cytoplasm of each cell. ***p< 0.001 **F** Nuclear and cytoplasmic protein extracts of DAOY-S and DAOY-R were analysed by western blot to evaluate NRF2 subcellular localization. As loading control, the blot was probed with HSP70 (cytoplasmic fraction) and Histone H3 (nuclear fraction). Western blot bands where quantified by densitometric analysis and normalized on the relative loading control. Data are expressed as the mean±SEM from 3 independent experiments. $*P < 0.05$ compared to DAOY-S.

Supplementary figure S3

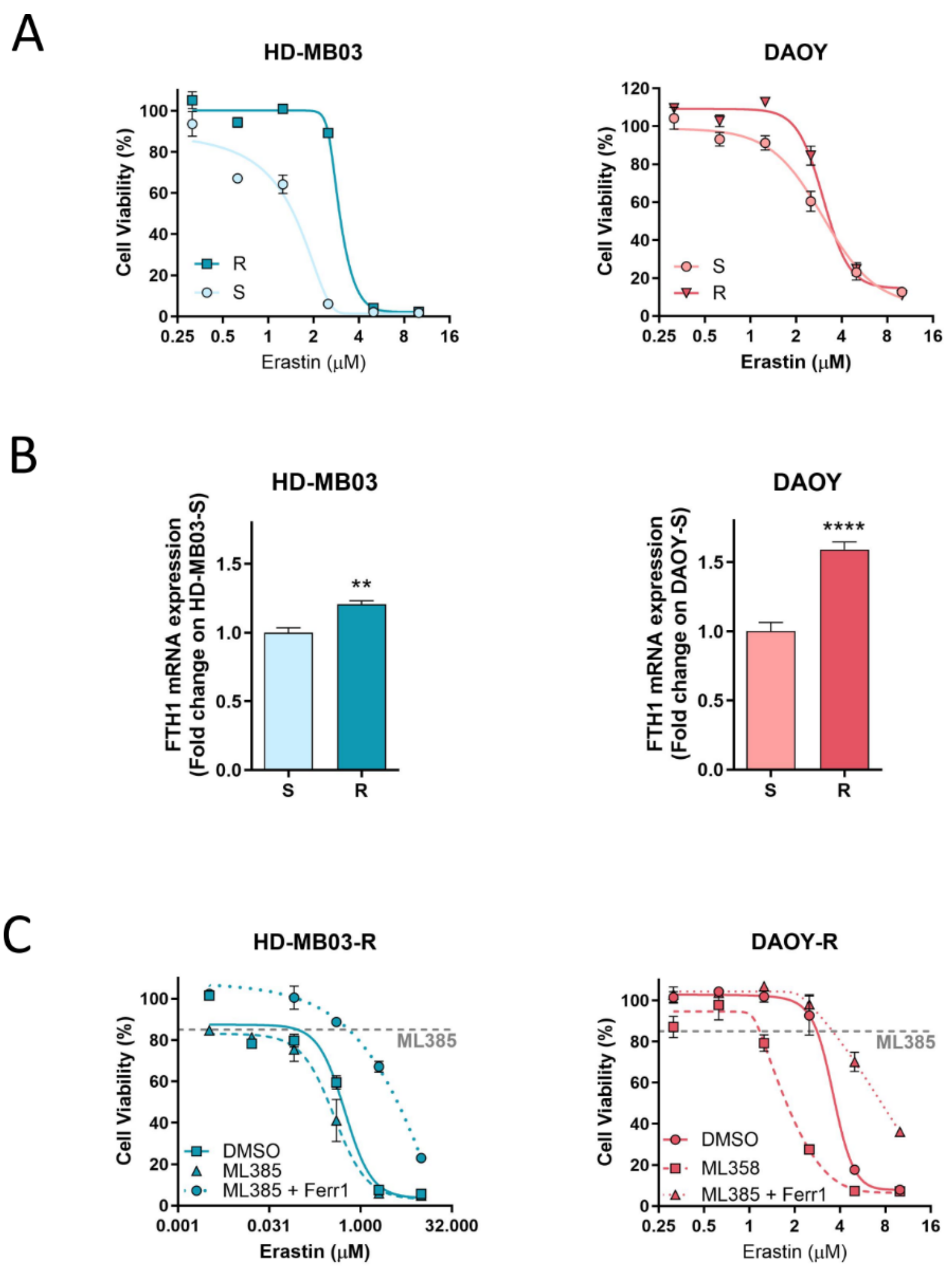

Fig S3 A Dose-response curve displaying increased resistance to Erastin treatment (72h) of MB-R models relative to their sensitive counterparts (MB-S). **B** FTH1 mRNA expression in MB-R and MB-S cells represented as the mean ± SEM of the fold change of FTH1 expression relative to MB-S cell lines. mRNA expression values were normalized to GUSB according to the ΔΔCt method. *$p<0.05$, **$p<0.01$. **C** Dose-response analysis of HD-MB03 and DAOY-R cells treated with Erastin alone and combined with the NRF2 inhibitor ML385 at the fixed concentration of 5μM and or Ferrostatin (Ferr1) at the fixed concentration of 1μM.

Supplementary figure S4

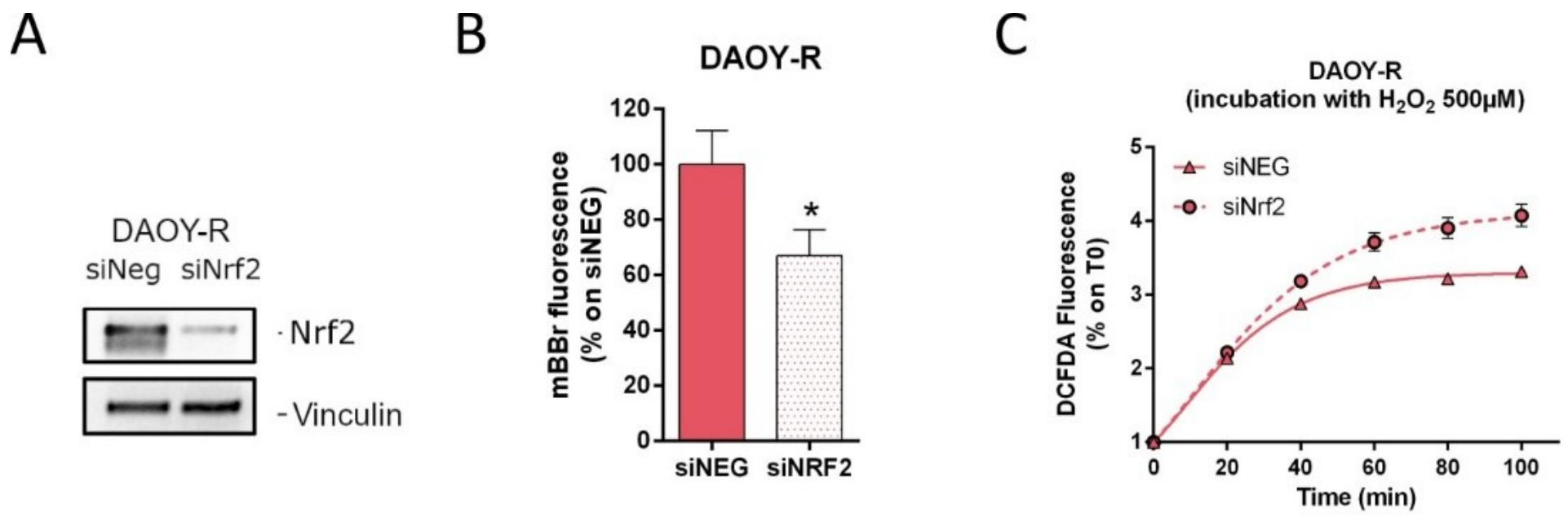

Fig S4 A Western Blot analysis on DAOY-R transfected with siNeg or siNrf2 displaying the expression of Nrf2 protein. **B** Flow cytometry analysis of GSH content in NRF2 KD cells using MBB staining. Data are represented as a percentage relative to the DAOY-R cells transfected with siNeg. **C** ROS levels during oxidative stress by incubation with hydrogen peroxide (500 μM) were measured over time (120 min) in DAOY-R-siNEG/siNRF2.

Supplementary figure S5

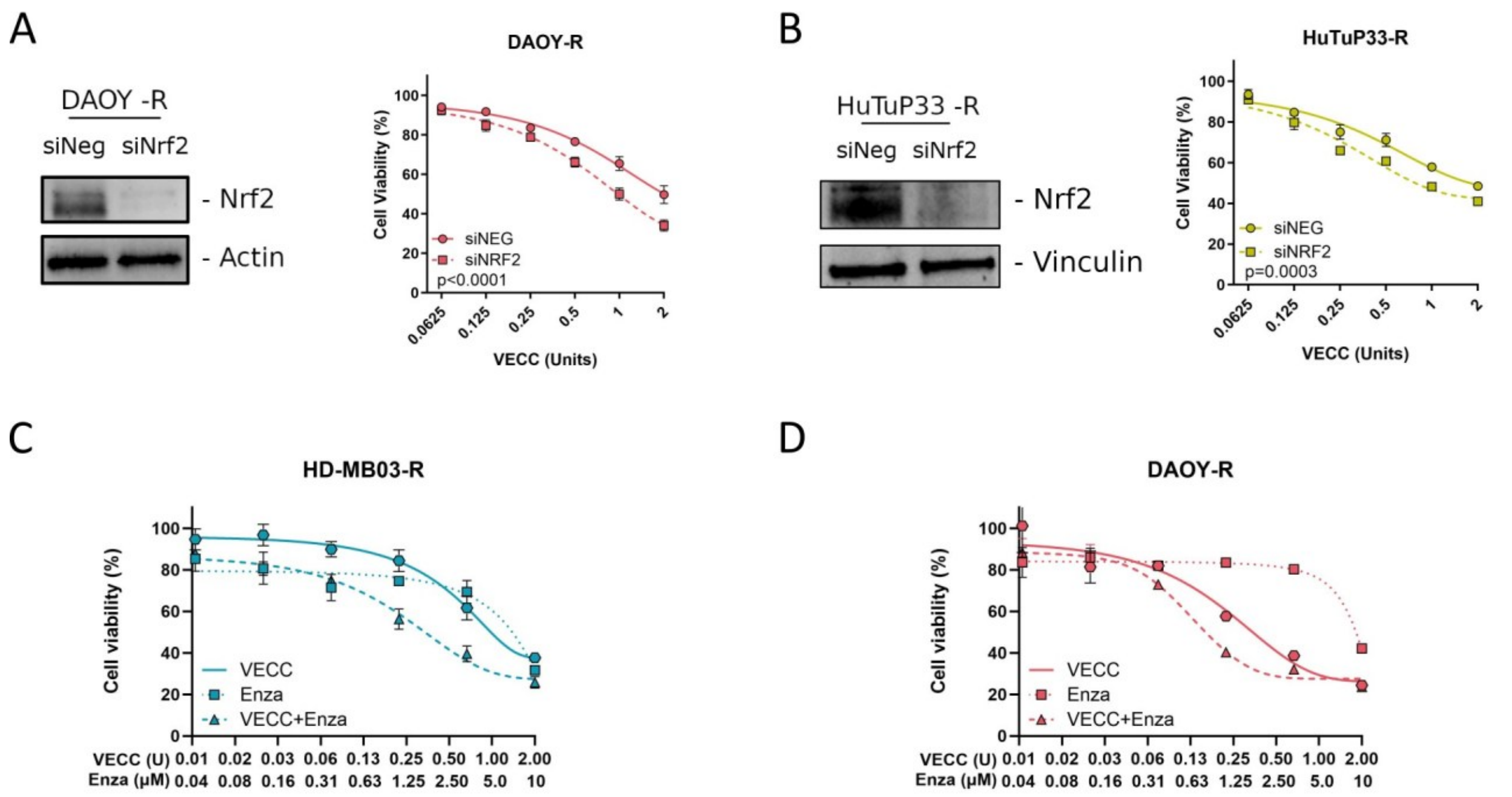

Fig S5 Dose-response analysis of HD-MB03 (**A**) and DAOY-R (**B**) cells treated with VECC, Enzastaurin, and their combination at fixed molar ratio, at the indicated doses. **C** Western blot analysis showing Nrf2 silencing in DAOY-R cells and VECC effect on cell viability DAOY-R cells after Nrf2 silencing. **D** Western blot analysis showing Nrf2 silencing in HuTuP33-R cells and VECC effect on cell viability HuTuP33-R cells after Nrf2 silencing.
